# Targeting VISTA as a novel immunotherapy for skin cancer

**DOI:** 10.3389/fimmu.2026.1815113

**Published:** 2026-09-17

**Authors:** Yulong Hou, Xi Chen, Can Chen

**Affiliations:** 1 Huzhou Central Hospital, Fifth School of Clinical Medicine of Zhejiang Chinese Medical University, Huzhou, China; 2 Huzhou Central Hospital, Affiliated Central Hospital of Huzhou University, Huzhou, China; 3 Department of Dermatology, First Affiliated Hospital of Huzhou University, Huzhou, China

**Keywords:** immunotherapy, pH-sensitive regulation, skin cancer, tumor microenvironment (TME), VISTA

## Abstract

While immune checkpoint blockade (ICB) targeting PD-1 and CTLA-4 has revolutionized the treatment of skin cancers, a significant proportion of patients with advanced melanoma and cutaneous squamous cell carcinoma (cSCC) exhibit primary or acquired resistance. Identifying non-redundant inhibitory pathways is crucial for the next generation of immunotherapy. As a unique checkpoint, VISTA (PD-1H) maintains immune tolerance through a distinct pH-sensitive mechanism. In the acidic tumor microenvironment (TME), protonated histidine residues enhance VISTA’s binding affinity, allowing it to act as a localized guardian of immune privilege in both melanoma and cutaneous squamous cell carcinoma. By integrating molecular insights with bioengineering innovations, VISTA-directed rational combinations demonstrate significant preclinical potential to overcome immune resistance and achieve durable responses in refractory malignancies.

## 1 Introduction

Skin cancer, including melanoma and non-melanoma types, poses a significant global health burden (1, 2). Despite advances in diagnosis and therapy, these malignancies—especially in advanced stages—remain a deadly threat with rising global incidence, underscoring the urgent need for more effective and tolerable systemic treatments (3).

The past decade has witnessed a fundamental shift in the paradigm of cancer therapy, with immunotherapy emerging as the fifth pillar of treatment, following surgery, radiotherapy, chemotherapy, and targeted therapy (4, 5). At the heart of this revolution lies the successful application of immune checkpoint blockade (ICB) therapy, which functions by relieving suppression of the host’s anti-tumor immune response (6). Inhibitors of the cytotoxic T-lymphocyte-associated protein 4 (CTLA-4) and programmed death receptor 1/programmed death ligand 1 (PD-1/PD-L1) pathways have demonstrated disease-modifying potential in various cancers, capable of inducing durable responses and even clinical cures (7, 8). In the field of skin cancer, particularly melanoma, immune checkpoint inhibitors have achieved particularly remarkable success. Long-term follow-up data from the CheckMate 067 trial showed that nivolumab combined with ipilimumab or nivolumab monotherapy for advanced melanoma provided significantly prolonged and durable survival benefits compared to ipilimumab monotherapy, with median overall survival not yet reached in the combination therapy group (8). This established immune combination therapy as the first-line standard-of-care for advanced melanoma, as recommended by American Society of Clinical Oncology (ASCO) guidelines (9). Similarly, in locally advanced and metastatic cutaneous squamous cell carcinoma (cSCC), immune checkpoint inhibitors targeting the PD-1/PD-L1 pathway have ushered in a therapeutic renaissance, offering patients a treatment option with lower toxicity and more durable responses compared to conventional chemotherapy (1).

However, ICB therapy is far from a panacea. Despite historic breakthroughs, only a subset of patients benefit from existing therapies, with a substantial proportion facing primary or acquired resistance (10, 11). In advanced melanoma, even with the most effective nivolumab plus ipilimumab regimen, a large number of patients fail to achieve long-term remission (12). This clinical challenge stems from the complex and dynamic interplay between tumors and the immune system. Tumors can evade immune clearance through multiple mechanisms, a process summarized as “cancer immunoediting,” wherein the immune system not only acts to suppress tumors but may also select more aggressive tumor clones under pressure or foster an immunosuppressive tumor microenvironment (TME), thereby promoting tumor progression (13). The TME is a complex ecosystem composed of diverse cell types, soluble factors, and physical barriers that profoundly influence the recruitment, survival, and function of effector T cells, constituting a major barrier to immunotherapy (14, 15). Existing ICB agents, particularly combination regimens, are often accompanied by significant and potentially life-threatening immune-related adverse events (irAEs), limiting their use in broader patient populations (12, 16). Therefore, gaining a deep understanding of the mechanisms underlying immune resistance and developing next-generation therapies to overcome these barriers are top priorities in current tumor immunology research (17, 18).

A central strategy to address these challenges is the identification and targeting of co-inhibitory immune checkpoint molecules beyond PD-1/PD-L1 and CTLA-4. A series of novel immunomodulatory receptors expressed in the TME provide a rich repository of targets for developing more effective and safer combination therapies (10, 17). Among these emerging targets, V-domain Ig suppressor of T cell activation (VISTA) has garnered significant attention due to its unique biological properties. VISTA is primarily expressed on myeloid cells and T cells, playing a critical role in maintaining immune homeostasis and tolerance (17, 19, 20). Growing evidence indicates that VISTA is highly expressed in the TME of various cancers, including melanoma, and is associated with poor prognosis and immune evasion (19–21). In contrast to classic checkpoints like PD-1, a notable feature of VISTA is its enhanced function in acidic microenvironments (e.g., hypoxic regions of tumors), a pH-dependent regulatory mechanism that makes it an ideal target for hostile TMEs (10). This uniqueness suggests that targeting VISTA may overcome specific immune resistance mechanisms driven by the tumor’s acidic microenvironment, thereby complementing existing therapies. Preliminary preclinical studies have shown that monoclonal antibodies targeting VISTA can enhance anti-tumor immune responses and may synergize with other immunotherapies (10). Furthermore, given its unique expression patterns and regulatory mechanisms, it is hypothesized that VISTA-targeted therapeutic strategies may also help mitigate the systemic toxicities associated with traditional ICB. For instance, experimental approaches involving local delivery systems or pH-responsive nanocarriers to specifically deliver drugs to tumor sites are currently being evaluated in preclinical settings (21).

To ensure a comprehensive and transparent evaluation of these emerging therapeutic strategies and the underlying biology of VISTA, a meticulous literature search was conducted across three primary electronic databases: PubMed, Web of Science, and Scopus. The search encompassed peer-reviewed articles published from database inception up to July 2026 and was restricted to the English language. The search strategy utilized combinations of keywords and Medical Subject Headings (MeSH) terms, including (“VISTA” OR “V-domain Ig suppressor of T cell activation” OR “PD-1H”) AND (“skin cancer” OR “melanoma” OR “cutaneous squamous cell carcinoma”) AND (“immunotherapy” OR “immune checkpoint” OR “tumor microenvironment”). By synthesizing the identified literature, this review aims to provide a robust and unbiased overview of VISTA’s unique molecular mechanisms, its dynamic role within the skin cancer microenvironment, and the promising trajectory of VISTA-targeted interventions.

## 2 The molecular biology, structure, and pH-sensitive regulation of VISTA

VISTA, also known as PD-1H, is a type I transmembrane protein belonging to the B7/CD28 immune checkpoint family, yet it exhibits limited homology to other members, conferring unique structural and functional attributes (22, 23). As originally delineated in the landmark discovery study by Wang et al., VISTA was initially identified through a genomic database search using the immunoglobulin variable (IgV) region of PD-1 and was recognized for its ability to suppress T cell-mediated immune responses (24). Unlike classical checkpoints such as PD-1 and CTLA-4, which are primarily induced upon T cell activation, VISTA is constitutively expressed on resting naïve T cells and across the myeloid lineage, including macrophages, dendritic cells, and neutrophils, positioning it as a critical homeostatic regulator of both innate and adaptive immunity (23, 25, 26). This foundational expression pattern underpins its distinct role in maintaining peripheral tolerance and modulating inflammatory responses, setting the stage for its complex involvement in cancer immunology.

The three-dimensional structure of the human VISTA extracellular domain, resolved at high resolution, reveals a canonical IgV-like fold but with several distinctive features that are central to its function (27, 28). A hallmark of VISTA’s structure is its elongated CC’ loop and an unusually high content of surface-exposed histidine residues clustered in the complementarity-determining region (CDR)-like proximal half of the molecule (27). These histidine clusters are functionally critical for VISTA’s coinhibitory activity. The protonation state of these histidines is sensitive to pH changes, a property that directly influences VISTA’s binding interactions. In the slightly acidic microenvironment typical of solid tumors, these histidine residues become protonated, enhancing VISTA’s binding affinity. This pH-sensitive switch is a key molecular mechanism that allows VISTA to exert potent immunosuppressive effects precisely within the acidic tumor niche (29) (**Figure 1**). Furthermore, VISTA’s IgV domain possesses a non-canonical topology, including an extra ‘H’ β-strand and a unique ‘clamping’ disulfide bond not found in other IgV-like structures, which likely restricts its orientation on the cell surface (27). Two additional disulfide bonds stabilize the core of the domain, contributing to its structural integrity and distinguishing it from other B7 family members (28).

**Figure 1:**
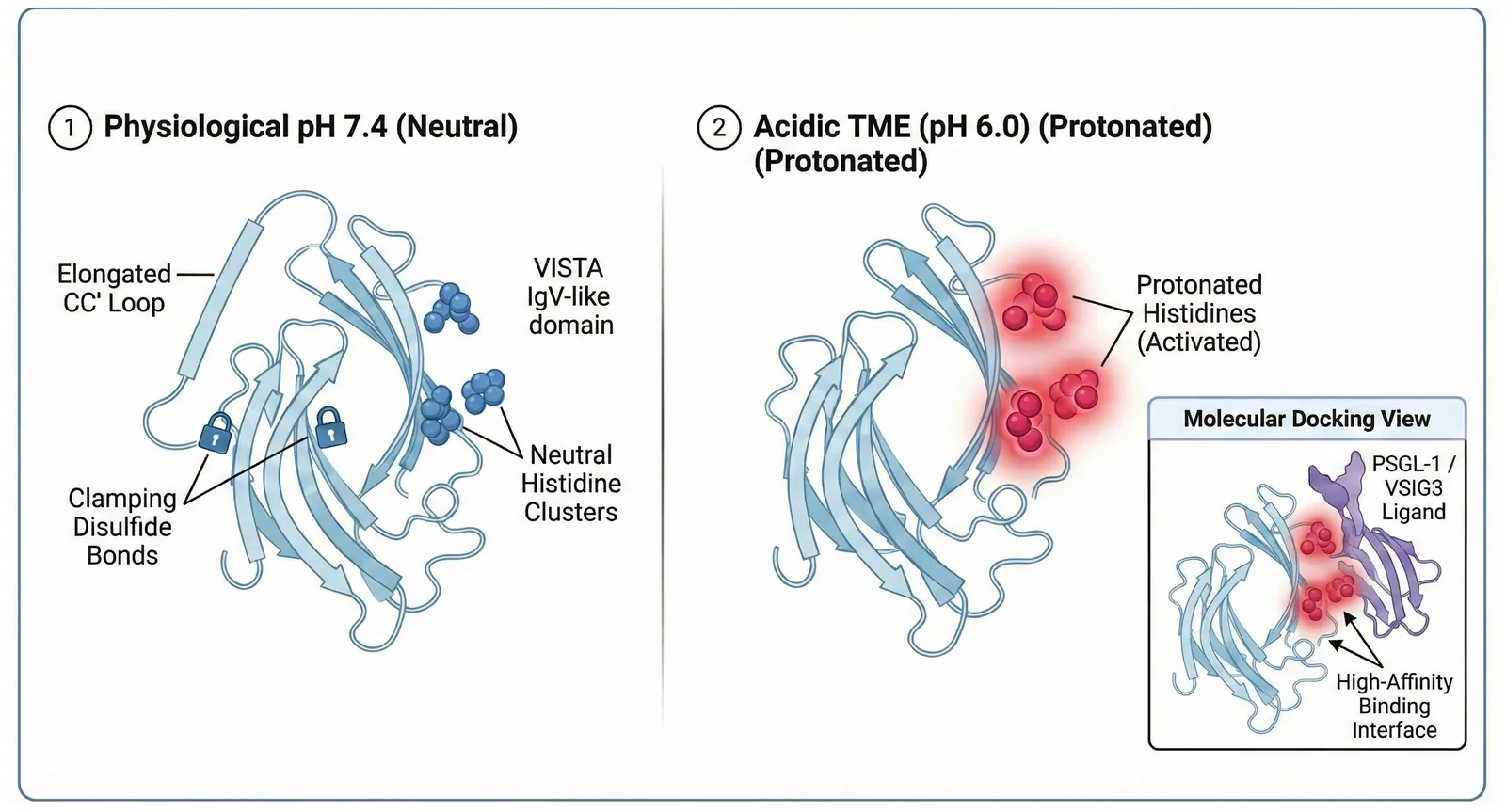
Molecular mechanism of VISTA as a pH-responsive immunological switch.

This unique structural arrangement defines the functional binding epitopes on VISTA. A contiguous binding region, encompassing the extended CC’ loop and an attached helix, forms the critical epitope for interaction with both functional binding partners and therapeutic antibodies (28). For instance, the binding site for V-Set and Immunoglobulin domain containing 3 (VSIG3), a putative receptor, overlaps with this region (28). The pH-dependent activity is strategically exploited by next-generation therapeutic agents. The pH-selective antibody SNS-101 is engineered to bind with high affinity to VISTA specifically in the acidic TME while showing minimal binding at physiological pH. This selectivity is achieved by targeting an epitope dependent on the protonation of key histidine residues, which confers a superior pharmacokinetic and safety profile by reducing on-target, off-tumor binding in healthy tissues (29). This rational design underscores the importance of understanding VISTA’s pH-sensitive interface for developing safer and more effective therapeutics.

The receptor-ligand landscape for VISTA is complex and context-dependent, involving both cis- and trans-interactions. VISTA can function as both a receptor on T cells and a ligand expressed on antigen-presenting cells or tumor cells (22, 30). Several binding partners have been proposed. P-selectin glycoprotein ligand-1 (PSGL-1) expressed on T cells can interact with VISTA. VSIG3 and VSIG-8 have also been identified as potential receptors or binding partners for VISTA (28, 31, 32). However, the physiological relevance of VSIG3 as a bona fide receptor remains highly controversial. Notably, Johnston et al. reported conflicting data, demonstrating that VISTA fails to bind to VSIG3 on the cell surface under any pH conditions (ranging from acidic to neutral). This critical finding questions the validity of initial biochemical reports and suggests that earlier observations might be artifacts of soluble protein assays rather than reflecting true cell-surface dynamics (33). Consequently, the field is increasingly shifting focus toward PSGL-1 and LRIG1 as the more definitive and physiologically relevant functional receptors for VISTA. The interaction between VISTA and VSIG-8 can suppress interleukin-2 (IL-2) production, and disruption of this interaction with a small molecule inhibitor has been shown to promote T cell cytokine production and inhibit melanoma growth in preclinical models (31). More recently, leucine-rich repeats and immunoglobulin-like domains 1 (LRIG1) was identified as a direct T cell-expressed binding partner for VISTA (34). The VISTA-LRIG1 interaction delivers an inhibitory signal that suppresses T cell receptor (TCR) signaling pathways. Genetic deletion of LRIG1 in T cells led to enhanced antitumor cytotoxic T lymphocyte (CTL) responses, characterized by expanded effector and memory-like populations, highlighting this axis as a critical downstream mechanism of VISTA-mediated suppression (34) (**Figure 2**). Additionally, extrapolating from research in other malignancies, VISTA can engage in heterophilic interactions, such as recognizing galectin-9 (Gal-9) secreted by tumor cells like acute myeloid leukemia blasts. While yet to be fully validated in the specific context of skin cancer, this VISTA-Gal-9 interaction can trigger intracellular granzyme B activation and apoptosis in cytotoxic T cells, representing a direct cytotoxic mechanism of immune evasion (35). The expression dynamics within the TME are interconnected, as evidenced in breast cancer where knockdown of one member of the VISTA/VSIG3/PSGL-1 axis upregulated the expression of the others, suggesting compensatory regulation (32).

**Figure 2:**
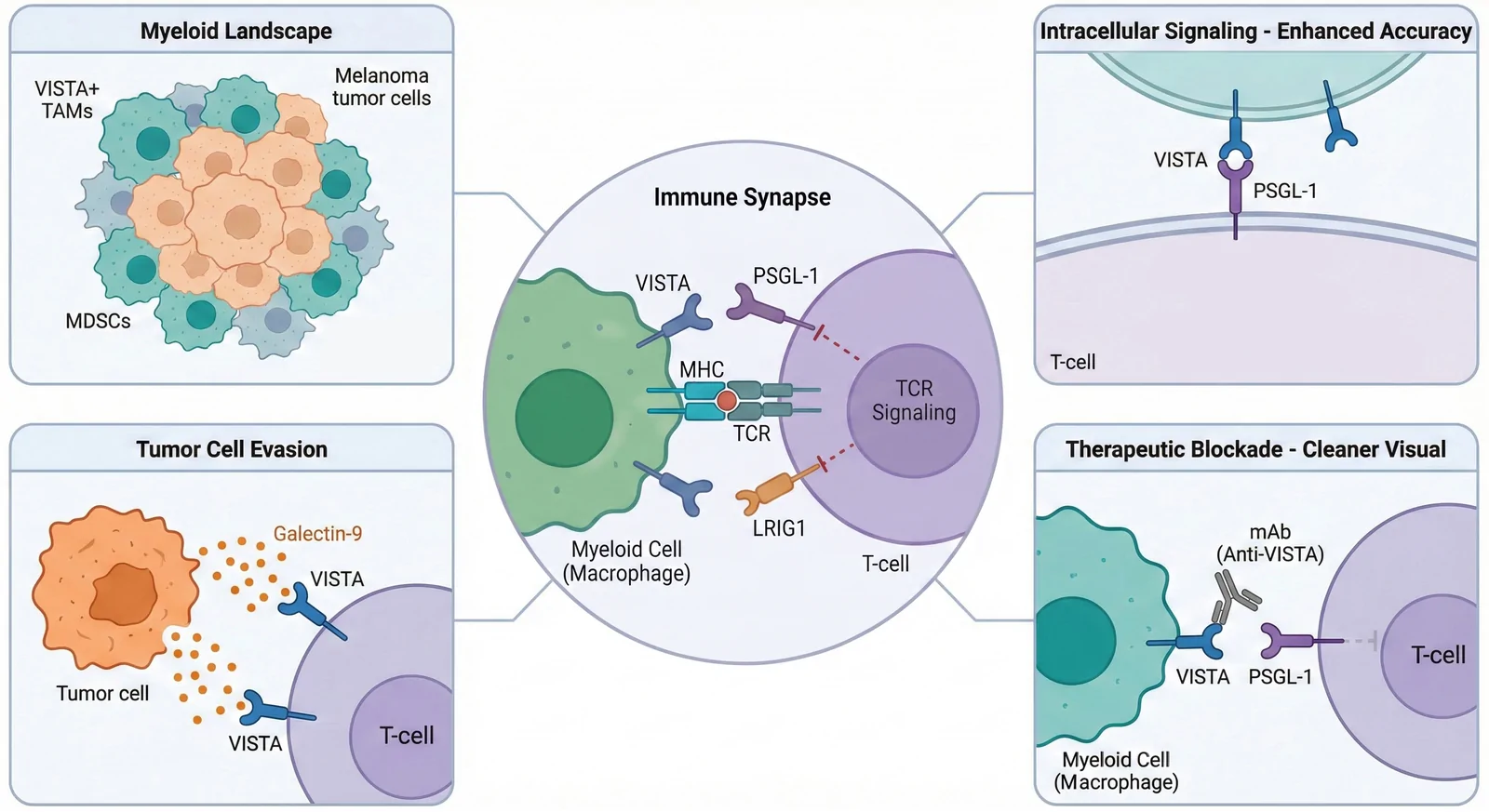
The multi-faceted interaction network of VISTA in immune suppression.

The expression of VISTA is regulated at multiple levels, including transcriptional, epigenetic, and metabolic pathways. In various cancers, including melanoma, ovarian, and endometrial cancers, VISTA expression on tumor cells themselves has been documented and linked to immune suppression (30, 36). In endometrial cancer, the methylation status of the VISTA promoter region influences its expression, pointing to epigenetic control (30). A significant regulatory axis involves the aryl hydrocarbon receptor (AHR). The AHR, a ligand-activated transcription factor, can be engaged by endogenous metabolites like kynurenine (a tryptophan catabolite) produced by tumor cells (37). Activation of this AHR pathway promotes VISTA expression. In melanoma cells, VISTA levels were shown to be under the control of AHR, as AHR knockdown or inhibition dramatically reduced VISTA expression (36). This link is therapeutically relevant, as the anti-diabetic drug metformin was found to suppress both AHR and VISTA expression in melanoma models (36, 38). Another metabolic regulator is aldehyde dehydrogenase 2 (ALDH2). ALDH2-mediated detoxification of endogenous aldehydes was found to sustain VISTA expression by activating the nucleotide oligomerization domain (NOD)/nuclear factor kappa-B (NF-κB) signaling pathway. Genetic or pharmacological inhibition of ALDH2 downregulated VISTA, revitalized CD8+ T cell function, and synergized with ICB (39).

Functionally, VISTA exerts its immunosuppressive effects through diverse mechanisms on different immune cell compartments. On naïve T cells, VISTA acts as a critical checkpoint for maintaining quiescence and peripheral tolerance. Loss of VISTA disrupts the quiescent naïve T cell pool, leads to accumulation of spontaneously activated T cells, and, in genetically susceptible backgrounds, dramatically enhances autoimmunity, indicating its non-redundant role in setting the threshold for T cell activation (25, 40). Its agonistic engagement can promote antigen-induced peripheral T cell deletion, reinforcing tolerance (25). On myeloid cells, VISTA functions as a potent regulator of innate inflammation. VISTA agonism can transcriptionally and epigenetically re-program macrophages towards a tolerant and anti-inflammatory phenotype, augmenting lipopolysaccharide (LPS) tolerance and reducing the production of pro-inflammatory cytokines like TNF and IL-6 (26). This dual role in restraining both naïve T cell responses and innate inflammation underscores VISTA’s function as a broad immune homeostat. However, under inflammatory conditions such as those in the TME, VISTA’s restraint on T cells can be overcome, yet its expression on myeloid cells and tumor cells continues to suppress antitumor immunity (22, 25). This multifaceted functionality necessitates precise therapeutic targeting strategies—antagonism to block its inhibitory signals in cancer versus agonism to suppress pathological inflammation in autoimmune settings (23, 41).

## 3 Expression patterns and functional roles of VISTA in the skin cancer TME

Building upon its unique molecular biology and pH-sensitive regulation, the expression of VISTA within the TME of skin cancers reveals a complex and context-dependent role in immune modulation. Its pattern of expression is not uniform across different skin cancer subtypes or even within individual tumors, reflecting the heterogeneous nature of the immune landscape. In cutaneous melanoma, VISTA expression is observed on both immune and tumor cells, a duality that informs its functional impact. Single-cell RNA sequencing of acral melanoma, a subtype with a notoriously immunosuppressive microenvironment, revealed that VISTA was expressed in a significant 58.3% of myeloid cells, highlighting its prominence on this immunosuppressive compartment (42). TIGIT was more associated with T/natural killer (NK) cells (42). This study also noted that acral melanomas were characterized by lower overall immune infiltrate and fewer effector CD8+ T cells compared to non-acral cutaneous melanomas, situating VISTA-expressing myeloid cells within a broadly suppressed immune context (42). Beyond immune cells, melanoma cell-intrinsic expression of VISTA is regulated by the stemness factor Forkhead box D3 (FOXD3) (43). BRAF inhibition upregulates FOXD3, which in turn transcriptionally represses VISTA, creating a dynamic link between oncogenic signaling pathways and checkpoint expression (43). Functionally, tumor cell-specific VISTA expression promotes tumor onset *in vivo*, correlating with increased intratumoral regulatory T cells (Tregs) and enhanced PD-L1 expression on tumor-infiltrating macrophages (43). This suggests that tumor-derived VISTA can sculpt the myeloid compartment towards a more immunosuppressive state.

In cSCC, another major non-melanoma skin cancer, VISTA expression presents a distinct profile. A study using multiplexed quantitative immunofluorescence found VISTA expressed in 48% of primary cSCCs, while PD-L1 was expressed in 58% (44). High VISTA expression correlated more strongly than PD-L1 with greater T-cell infiltration and activation, as measured by Ki-67 and granzyme B (44). There was no significant correlation between VISTA and PD-L1 co-expression within the same tumors, indicating that individual cSCCs may employ non-redundant, distinct immunosuppressive mechanisms (44). This decoupling suggests that VISTA might define an immunosuppressive axis independent of the PD-1/PD-L1 pathway, a finding with direct implications for patient stratification and combination therapy. The uveal melanoma (UM) landscape offers another perspective. In this aggressive ocular melanoma, VISTA expression, analyzed via The Cancer Genome Atlas (TCGA) and immunohistochemistry, was associated with tumor aggressiveness, T cell exhaustion, and the shortest median overall survival (45). PD-1 protein was negative in all patient samples, while CTLA-4 expression was high in advanced stages, positioning VISTA as a potentially dominant and prognostically negative immune checkpoint in this immunologically “cold” tumor (45). A separate study on primary UM found VISTA protein expressed specifically in small tumor-infiltrating lymphocytes (TILs), not tumor cells, and its higher expression in TILs was associated with better prognosis and nuclear BAP1 expression (46). This apparent contradiction—VISTA linked to both poor and favorable outcomes—underscores the critical importance of defining the cellular source (tumor vs. immune cell) and the broader immunological context when interpreting VISTA’s role, a theme observed in other cancers like endometrial carcinoma where immune cell VISTA correlated with improved survival (47).

To critically analyze these prognostic discrepancies, several underlying biological mechanisms must be considered. First and foremost is the divergence based on the cellular source of expression. Tumor-cell-intrinsic VISTA acts primarily as a direct structural ligand for immune evasion, actively shielding the tumor from cytotoxic attack, which typically drives tumor progression and correlates with poor survival. Conversely, VISTA expression on the immune infiltrate (such as TILs or macrophages) often reflects a reactive, inflamed TME. Similar to the well-established ‘adaptive resistance’ paradigm of PD-L1—where its upregulation is triggered by local immune activation and IFN-γ production—high VISTA levels on immune cells may serve as a surrogate marker for a robust, pre-existing anti-tumor immune response. Therefore, patients with high immune-VISTA might paradoxically exhibit better baseline survival than those with immunologically ‘cold’ tumors lacking VISTA and T-cell infiltrates entirely. Second, VISTA’s unique functional duality as both an inhibitory receptor on T cells and a suppressive ligand on antigen-presenting cells introduces highly complex, bidirectional signaling networks. The net prognostic outcome in any given tumor likely depends on the stoichiometric balance and spatial availability of its specific binding partners, such as LRIG1, PSGL-1, or VSIG-3, within localized tumor niches. Finally, the temporal dynamics of expression further confound bulk prognostic correlations. VISTA may be constitutively expressed early in tumorigenesis to establish tolerance, or it may be dynamically upregulated as a late-stage compensatory mechanism following the failure of primary immune responses. Consequently, understanding VISTA’s true prognostic value necessitates moving beyond simple bulk tissue transcriptomics towards high-resolution, spatially preserved, and cell-type-specific profiling.

The cellular distribution of VISTA across disease stages in melanoma has been systematically mapped. A comprehensive multiplex immunofluorescence study of treatment-naïve patients found that checkpoint receptors like TIM-3 and VISTA were most abundant on non-T cells within the TME (48). Tumor-resident CD8+ T cells (CD69+/CD103+) were highly enriched for T cell immunoreceptor with Ig and ITIM domains (TIGIT) but not for the costimulatory receptors GITR or OX40 (48). In cSCC, OX40 is predominantly expressed on suppressive Tregs (49). The proportion of GITR+ T cells decreased from primary melanoma to metastases, whereas VISTA’s expression pattern on myeloid cells appeared more consistent (48). This highlights VISTA as a checkpoint predominantly borne by the innate immune compartment, particularly myeloid-derived suppressor cells (MDSCs) and macrophages, throughout melanoma progression (**Figure 3**). The mechanistic underpinnings of this myeloid-associated suppression are profound. Xu et al. elegantly demonstrated that VISTA impairs Toll-like receptor (TLR) signaling partly through controlling TRAF6 turnover, and that blocking VISTA synergizes with TLR-agonistic vaccines to eradicate tumors in murine melanoma models (50). Furthermore, VISTA actively promotes the development of MDSCs; crucially, the conditional deletion of VISTA specifically within monocytic progenitors is sufficient to control tumor growth in murine melanoma models, confirming its indispensable role in myeloid-driven immune evasion (51). The immunosuppressive function of these VISTA+ myeloid populations is often augmented by other metabolic and checkpoint pathways. CD71+ erythroid cells (CECs), which expand in cancer and correlate with poor response to PD-L1 blockade, exhibit a robust immunosuppressive phenotype characterized by expression of reactive oxygen species, PD-L1/PD-L2, and VISTA (52). In the adenosine-rich TME, CD39 and CD73 work in tandem to convert extracellular ATP to immunosuppressive adenosine, which signals through the A2A receptor on T cells to impair their effector functions and metabolic fitness (53–55). Adenosine limits the efficacy of anti-CTLA-4 therapy, and its blockade synergizes with checkpoint inhibition in melanoma models (56). This adenosinergic pathway is active in melanoma, with adenosine produced by cells including Tregs, MDSCs, and activated effector T cells (57). This creates a layered immunosuppressive network where VISTA expression on myeloid cells may coincide with adenosinergic and other inhibitory signals.

**Figure 3:**
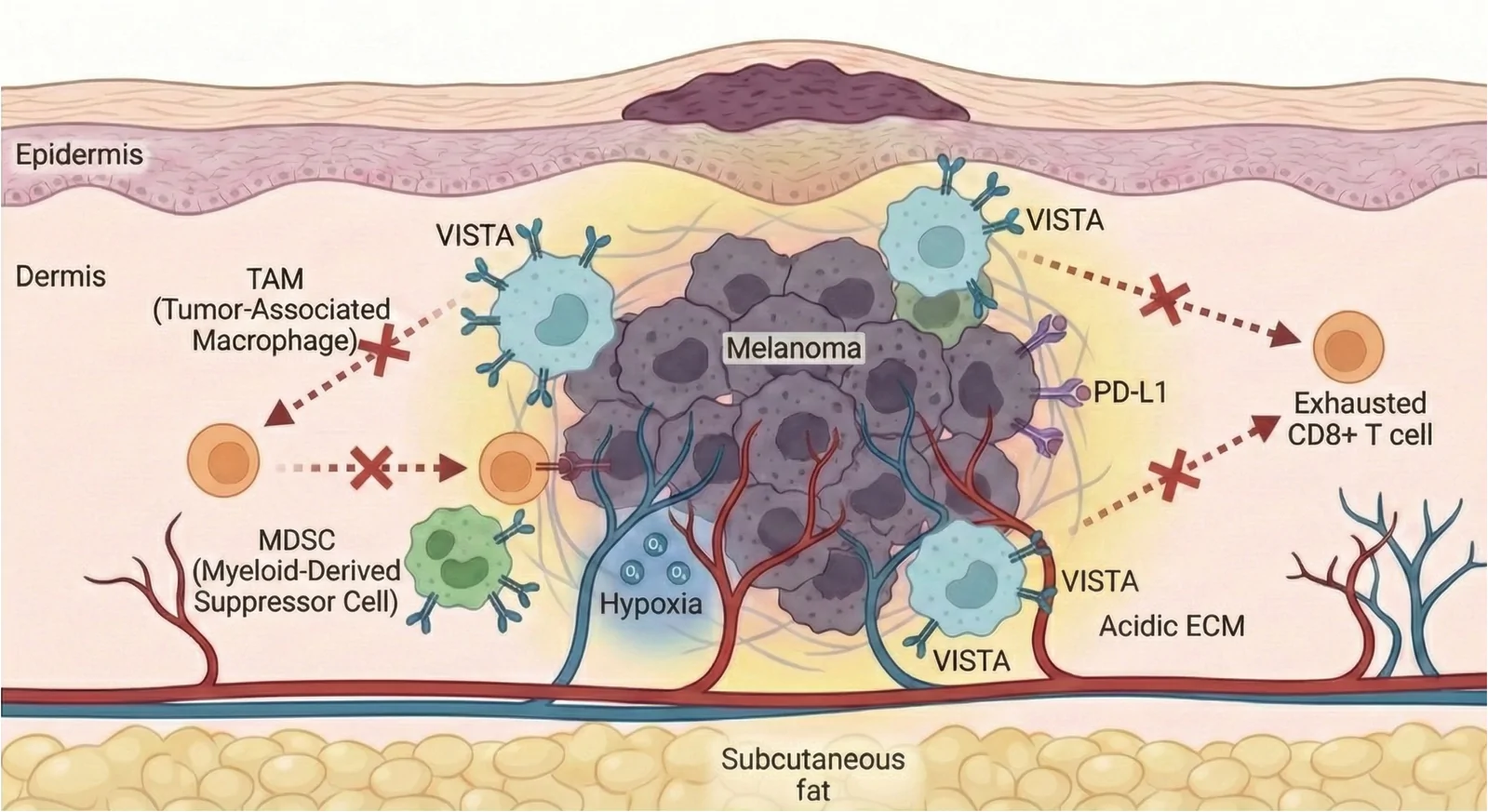
Spatiotemporal expression profile of VISTA in the skin cancer microenvironment.

VISTA’s functional role is deeply intertwined with the spatial organization and functional state of T cells in the TME. High VISTA expression in cSCC correlates with T cell infiltration and activation, yet this may represent an “adaptive resistance” mechanism similar to that described for PD-L1, where immune attack triggers local immunosuppressive feedback (44, 58). In melanoma, the coexistence of tumor antigen-specific T cells and progressive disease—the “Hellstrom paradox”—can be explained by such mechanisms, where the TME actively suppresses infiltrating T cells (55). VISTA contributes to this by directly inhibiting T cell activation. The functional suppression of T cells is often mediated by multiple, co-expressed inhibitory receptors. Exhausted T cells in melanoma can co-upregulate PD-1 and T-cell immunoglobulin and mucin-domain containing-3 (TIM-3), and dual blockade is more effective than targeting either alone (59). PD-1 and LAG-3 show extensive co-expression on TILs and synergistically regulate T-cell function to promote tumoral immune escape (60). VISTA’s expression on antigen-presenting cells allows it to suppress T cell responses in *trans*. The ligand-receptor interaction for VISTA is complex; VSIG-3/IGSF11 has been identified as an inhibitory ligand. In melanoma, silencing VSIG-3 in tumor cells not only suppressed tumor cell progression but also skewed co-cultured T cells towards a pro-inflammatory cytokine profile (increased IFN-γ, IL-12; decreased IL-10, TGF-β), indicating that the VISTA-VSIG-3 axis is an active modulator of T cell function within the TME (61).

The impact of VISTA extends beyond lymphocyte interactions to influence the broader stromal and metabolic milieu. Tumor-associated macrophages (TAMs), major players in the TME, can express VISTA and other checkpoints like PD-L1 (62). PD-L1 signaling in macrophages themselves exerts a constitutive negative signal, maintaining an immunosuppressive phenotype; antibody blockade can trigger macrophage re-activation and antitumor activity (63). The specific cellular source of checkpoint molecules matters: in lung cancer, PD-L1 expression on macrophages, not tumor cells, was associated with better survival after immunotherapy, suggesting that macrophage PD-L1 may be a marker of a pre-existing, though restrained, immune response (64, 65). Similar nuanced interpretations may apply to VISTA. Oncogenic pathways within tumor cells can dictate the immune contexture. Melanoma-intrinsic activation of the WNT/β-catenin pathway leads to T-cell exclusion and resistance to anti-PD-1/CTLA-4 therapy, creating a non-inflamed “immune desert” (66, 67). An epithelial-mesenchymal transition (EMT) phenotype in lung adenocarcinoma is associated with an inflammatory TME enriched for multiple immune checkpoints, including VISTA, suggesting that EMT may be a predictor for responsiveness to checkpoint blockade (68). In melanoma, resistance to anti-PD-1 therapy is characterized by an innate anti-PD-1 resistance (IPRES) signature encompassing genes involved in mesenchymal transition, wound healing, and angiogenesis (69). **Table 1** summarizes the diverse expression patterns, cellular localization, and functional implications of VISTA across cutaneous, acral, and uveal melanoma, as well as cSCC.

**Table 1:** Summary of VISTA expression patterns and clinical implications across skin cancer subtypes.

Skin cancer subtype	Primary cellular source	Key TME features	Clinical & functional implications
Cutaneous Melanoma (Non-acral)	Tumor cells, Myeloid cells (TAMs, MDSCs)	Highly abundant on non-T cells Tumor-intrinsic expression regulated by FOXD3	Promotes tumor onset Correlates with increased Tregs and enhanced PD-L1 on TAMs
Acral Melanoma	Myeloid cells	Expressed on 58.3% of myeloid cells. Associated with low overall immune infiltrate and few effector CD8+ T cells	Acts as a primary driver of immune privilege in a deeply suppressed microenvironment.
Uveal Melanoma (UM)	Tumor cells, TILs (Tumor-infiltrating lymphocytes)	Often emerges as the dominant checkpoint (PD-1 is frequently negative)	Context-dependent: - High overall expression: Shorter survival and T-cell exhaustion - Expression on small TILs: Better prognosis
Cutaneous Squamous Cell Carcinoma (cSCC)	Immune infiltrate	Expressed in 48% of primary tumors Lacks correlation with PD-L1 co-expression	Correlates with *greater* T-cell infiltration and activation Suggests a distinct, independent mechanism of adaptive resistance

TAMs, tumor-associated macrophages; MDSCs, myeloid-derived suppressor cells; Tregs, regulatory T cells; TILs, tumor-infiltrating lymphocytes; UM, uveal melanoma; cSCC, cutaneous squamous cell carcinoma.

In summary, VISTA occupies a unique and multifaceted niche in the skin cancer TME. Its expression on a high proportion of immunosuppressive myeloid cells, particularly in resistant subtypes like acral and uveal melanoma, positions it as a key guardian of immune privilege. Its expression on tumor cells, regulated by factors like FOXD3, adds an autonomous layer of immunosuppression. The lack of strong correlation with PD-L1 in cSCC suggests it defines a non-redundant resistance pathway. Its functional output—whether associated with poor prognosis as in UM or with T cell infiltration as in cSCC—is exquisitely context-dependent, hinging on the cellular source, the concurrent immune cell infiltrate, and the activation state of other complementary immunosuppressive networks like adenosinergic signaling. This complex expression pattern and functional versatility make VISTA a compelling but challenging therapeutic target, necessitating careful patient selection and rational combination strategies to effectively modulate its activity in the hostile skin cancer microenvironment.

## 4 The interplay between VISTA and other immune pathways: implications for resistance and rational combinations

The functional impact of VISTA is not isolated but is intricately woven into a broader tapestry of immunosuppressive signals within the TME. This network comprises both adaptive and innate immune checkpoints, metabolic pathways, and soluble mediators, which collectively sculpt an immune-resistant niche. Understanding the interplay between VISTA and these complementary pathways is critical for deciphering primary and acquired resistance to existing immunotherapies and for designing rational, synergistic combination strategies. In skin cancers, particularly melanoma and cSCC, the co-expression and functional cooperation of multiple inhibitory receptors on T cells and their ligands on tumor and myeloid cells represent a formidable barrier to monotherapy with agents targeting PD-1 or CTLA-4 (10, 70, 71). The compensatory upregulation of alternative checkpoints following single-agent blockade is a well-documented mechanism of adaptive resistance, highlighting the need for combinatorial approaches (72, 73).

VISTA engages in direct and indirect crosstalk with other key immune checkpoints. The foundational proof of concept for dual targeting was established by Liu et al., who first demonstrated the critical non-redundancy and cooperation between the VISTA and PD-1 pathways, showing that blocking both axes synergistically controlled melanoma growth in murine models (74). Its expression pattern often complements rather than mirrors that of PD-L1. As observed in cSCC, this lack of spatial correlation confirms that VISTA drives an independent immunosuppressive axis (75), providing a strong rationale for dual targeting. VISTA’s function can be modulated by upstream signaling pathways shared with other immunosuppressive molecules. As previously discussed, the ALDH2-NOD/NF-κB signaling axis serves as a direct mechanistic link between tumor metabolism and VISTA expression, positioning it as a metabolic-immune checkpoint circuit that can be targeted to synergize with PD-1 blockade (39). VISTA also functionally intersects with the galectin-9/TIM-3 pathway. Research in acute myeloid leukemia demonstrated that VISTA expressed on T cells can recognize galectin-9, a ligand for TIM-3, secreted by tumor cells. This interaction, potentially forming multiprotein complexes on the T cell surface, induces apoptosis of cytotoxic T cells, revealing a cooperative mechanism between these two pathways to suppress immunity (35). The relevance in melanoma is underscored by the established role of TIM-3 upregulation in T cell exhaustion and resistance to PD-1 blockade (59, 73, 76). The synergistic effect of dual PD-1 and TIM-3 blockade has been demonstrated in melanoma models, and combining a MEK inhibitor (trametinib) with anti-TIM-3 antibody also showed enhanced efficacy, partly because MEK inhibition paradoxically increased Tim-3 expression (77). This suggests that targeted therapies can reshape the checkpoint landscape, potentially inducing dependencies on pathways like VISTA.

The collaboration between VISTA and the PD-1/PD-L1 and LAG-3 pathways is of paramount clinical relevance. LAG-3 has emerged as the third validated immune checkpoint in melanoma following the success of relatlimab combined with nivolumab (12). Like VISTA, LAG-3 is frequently co-expressed with PD-1 on exhausted T cells within tumors. Preclinical studies have shown that PD-1 and LAG-3 synergistically regulate T-cell function to promote tumoral immune escape, with dual genetic knockout or antibody blockade curing mice of tumors resistant to single-agent therapy (60). Mechanistically, LAG-3 binding to stable peptide-MHC class II complexes, rather than other proposed ligands, is crucial for its suppressive activity in autoimmunity and anti-cancer immunity (78). The compensatory upregulation of LAG-3 and CTLA-4 has been observed as a mechanism limiting the efficacy of single-agent PD-1 blockade in other cancers, a phenomenon likely relevant to skin cancers (72). This biology has been translated into clinical tools, with bispecific antibodies targeting both PD-L1 and LAG-3 (e.g., IBI323) showing superior preclinical antitumor activity compared to parental antibodies or their combination, mediated by enhanced T cell responses (79). Given that VISTA and LAG-3 are both expressed on immunosuppressive myeloid populations and exhausted T cells, their concurrent blockade with PD-1 inhibition represents a logical, multi-pronged theoretical strategy to overcome a broader spectrum of resistance mechanisms that warrants future clinical exploration.

Beyond receptor-ligand checkpoints, VISTA operates within a milieu dominated by soluble immunosuppressive factors, most notably the adenosinergic pathway and TGF-β. Extracellular adenosine, produced by the ectoenzymes CD39 and CD73 on regulatory T cells (Tregs), myeloid-derived suppressor cells (MDSCs), and even activated effector T cells, is a potent immunosuppressor in melanoma (57). Signaling through the A2A adenosine receptor (A2AR) on T cells directly inhibits their activation and, importantly, also expands and enhances the function of immunosuppressive Tregs (56, 80). This pathway actively limits the efficacy of anti-CTLA-4 therapy, and its blockade synergizes with both anti-CTLA-4 and anti-PD-L1 (56, 81). The interconnection with VISTA is implied by the shared cellular sources and the overall immunosuppressive phenotype they create. Similarly, the TGF-β signaling pathway is a master regulator of immune exclusion, creating a fibroblast-rich stromal barrier that prevents T cell infiltration into tumor parenchyma. This phenotype is associated with resistance to PD-L1 blockade across cancers, including melanoma, and combined blockade of TGF-β and PD-L1 can overcome this resistance (82). While a direct link with VISTA is not yet established, TGF-β is a known driver of immunosuppressive myeloid and fibroblast states, cell types that frequently express VISTA, suggesting potential co-regulation within a resistant TME characterized by myeloid infiltration and stromal desmoplasia.

This complex network of interacting pathways directly informs mechanisms of resistance to current immunotherapies. Primary resistance can be attributed to a pre-existing TME rich in multiple inhibitory signals (VISTA, LAG-3, adenosine, TGF-β) that overwhelm initial T cell activation even after PD-1 or CTLA-4 blockade (70, 83). Acquired resistance, observed in patients who initially respond but later relapse, can occur through tumor-intrinsic mechanisms such as loss of antigen presentation (B2M mutations) or interferon signaling defects (JAK1/2 mutations), as documented in melanoma patients on anti-PD-1 therapy (84). However, immune-extrinsic adaptive resistance is equally critical. Upon selective pressure from PD-1/PD-L1 blockade, tumors and their microenvironment can upregulate alternative inhibitory pathways. Drawing parallels from lung cancer models and patients, where adaptive resistance to PD-1 blockade was associated with upregulation of TIM-3 on T cells (73). It is plausible that VISTA, given its role as a pH-sensing guardian of immune tolerance in acidic, inflamed sites like tumors, could be similarly upregulated as a compensatory resistance mechanism in melanoma and cSCC following effective but incomplete immune reactivation by first-line checkpoint inhibitors.

Therefore, the rational design of combination therapies must account for this interconnected biology. Combinations can be broadly categorized into vertical strategies targeting multiple steps in the immune cycle or horizontal strategies targeting parallel inhibitory pathways. For skin cancers, several rational combinations emerge from the interplay with VISTA. First, dual or triple ICB targeting VISTA alongside PD-1 and/or LAG-3 or CTLA-4 is a direct approach to overcome co-expression and redundancy (10, 71). Recent breakthroughs have also elucidated the critical role of T cell-intrinsic VISTA. Gilmour et al. demonstrated that blocking T cell-intrinsic VISTA synergizes potently with CTLA-4 inhibitors to boost tumor control in murine melanoma models. Importantly, they identified a T cell-intrinsic VISTA signature that correlates with clinical responses in human melanoma patients, solidifying the translational rationale for this specific combination (85). The success of nivolumab/ipilimumab and nivolumab/relatlimab sets a precedent for such combinations, though managing toxicity remains a challenge (12, 86, 87). Second, combining VISTA inhibition with therapies that target the immunosuppressive metabolic environment is compelling. This includes inhibitors of the adenosine pathway (A2AR antagonists, CD73 inhibitors) or indoleamine 2,3-dioxygenase (IDO), both of which have shown synergy with PD-1/CTLA-4 blockade in melanoma models and early-phase trials (56, 81, 88, 89). The ALDH2-VISTA axis provides another specific metabolic target for combination (39). Third, integrating VISTA blockade with agents that remodel the TME and enhance T cell infiltration is rational. This includes co-stimulatory agonists (e.g., anti-4-1BB, anti-OX40, anti-CD27) which have shown potential in preclinical melanoma and cSCC models to enhance effector function, reduce Treg suppression, and synergize with checkpoint blockade (49, 75, 90–92). Oncolytic viruses, cytokines, and therapies that normalize tumor vasculature or disrupt stromal barriers (e.g., TGF-β inhibitors) also fit this category (93, 94). Fourth, the combination with targeted therapies, such as BRAF/MEK inhibitors in melanoma, is crucial given their widespread use. As seen with trametinib increasing TIM-3, targeted agents modulate the immune landscape, potentially creating new dependencies on checkpoints like VISTA that can be therapeutically exploited (77). Finally, in experimental models, innovative delivery platforms such as pH-sensitive nanocarriers or microneedle patches for transcutaneous delivery can facilitate the local, sustained, and synergistic release of VISTA-targeting agents alongside chemotherapy or other immunomodulators. While currently confined to preclinical evaluations, this represents a particularly attractive theoretical approach for maximizing intratumoral efficacy while minimizing systemic toxicity in accessible skin cancers (89, 95).

In summary, VISTA does not function in isolation but is a pivotal node within a dense network of complementary immunosuppressive pathways in the skin cancer TME. Its interactions with PD-1, LAG-3, TIM-3, adenosinergic signaling, and metabolic circuits underpin both *de novo* and adaptive resistance to single-agent immunotherapy. Consequently, effective therapeutic targeting of VISTA will almost certainly require rational combination strategies. These strategies must be guided by a deep understanding of the dominant resistance mechanisms within a given patient’s tumor, which may be elucidated through biomarker analysis of the TME, including the co-expression patterns of multiple checkpoints, the presence of specific metabolic enzymes, and the activity of stromal remodeling pathways. The future of skin cancer immunotherapy lies in moving beyond monolithic approaches towards personalized combinations that simultaneously disarm multiple facets of the tumor’s immunosuppressive arsenal, with VISTA representing a key and unique component of this next-generation arsenal.

## 5 Therapeutic targeting of VISTA: from pH-selective antibodies and small molecules to nanomedicine and local delivery

Recognizing VISTA as a critical and complementary immune checkpoint within the complex immunosuppressive network of the skin cancer TME necessitates the development of specific therapeutic modalities to disarm it. Its established role in mediating resistance to established immunotherapies, such as anti-PD-1, underscores the urgency for effective VISTA-targeted agents (96). The therapeutic arsenal against VISTA is rapidly evolving. While early clinical trials are investigating systemic antibodies, a spectrum of preclinical strategies, ranging from small molecules to advanced nanomedicine and localized delivery systems, are currently under development. These experimental approaches aim to block its immunosuppressive signaling, modulate its function, or disrupt its interactions within the acidic TME, potentially offering new avenues to overcome immunotherapy resistance in melanoma and other cutaneous malignancies.

The foundational approach involves monoclonal antibodies designed to block VISTA’s interaction with its putative receptors, such as VSIG-8 or PSGL-1. Early work demonstrated that an anti-PD-1H (VISTA) monoclonal antibody could prevent acute graft-versus-host disease in murine models, highlighting its potent immunomodulatory potential (97). Subsequent engineering efforts have produced antibodies with enhanced properties. The antibody SG7 was developed to bind with high affinity to mouse, human, and cynomolgus monkey VISTA, inhibiting its function by blocking interactions with both PSGL-1 and VSIG3 (98). As a monotherapy and particularly in combination with anti-PD-1, SG7 demonstrated efficacy in slowing tumor growth across multiple syngeneic models (98). A significant advancement came with the development of pH-selective antibodies. Leveraging the distinct pH-dependent conformational changes described previously (27), the pH-selective antibody SNS-101 was engineered to bind VISTA with high affinity specifically under acidic TME conditions, while exhibiting markedly reduced binding at physiological pH (29). This selectivity confers a superior pharmacokinetic and safety profile by minimizing rapid clearance and, critically, avoiding cytokine release syndrome—a toxicity observed with earlier non-selective VISTA antibodies. In preclinical models using human VISTA knock-in mice, SNS-101 enhanced the antitumor response of PD-1 inhibition, demonstrating its promise as a combination agent (29). Another clinical candidate, KVA12123, is a fully human monoclonal antibody that binds a distinct VISTA epitope, blocks its interactions, and demonstrates single-agent and combination anti-PD-1 activity in syngeneic models, with a favorable safety profile in non-human primates (99).

Complementing biologic approaches, small molecules offer an alternative strategy for modulating the VISTA pathway, potentially with advantages in oral bioavailability and tissue penetration. The discovery of small molecules targeting this pathway is still nascent but promising. Compound M351–0056 was identified through virtual screening as a ligand for the human VISTA extracellular domain (100). It functioned as an immune-modulating agent by decreasing cytokine secretion, suppressing peripheral blood mononuclear cells (PBMC) proliferation, and enhancing Foxp3+ T cell numbers *in vitro*, effects mediated through the JAK2-STAT2 pathway. *In vivo*, M351–0056 ameliorated imiquimod-induced psoriasis-like dermatitis, suggesting therapeutic potential in inflammatory and autoimmune contexts (100). In a distinct approach targeting the VISTA interaction partner, the small molecule L557–0155 was developed as an inhibitor of VSIG-8, preventing its binding to VISTA (31). This inhibitor promoted cytokine production and cell proliferation in peripheral blood mononuclear cells and suppressed melanoma growth *in vivo*, validating the VSIG-8/VISTA axis as a druggable target for cancer immunotherapy (31). While most efforts focus on antagonists, engineered protein constructs like VISTA.COMP, a stable pentameric form of the VISTA IgV domain, have been designed to act as potent agonists for suppressing T cell-mediated responses, showcasing the pathway’s modulability for applications in autoimmunity or transplantation (41).

The limitations of systemic immunotherapy, including inefficient drug infiltration into tumors, high biodistribution to healthy tissues causing irAEs, and the development of resistance, have spurred innovation in drug delivery systems (DDS) (21, 101). Nanomedicine platforms are particularly suited to address these challenges by enabling targeted co-delivery of multiple therapeutic payloads to the TME (102). A prime example demonstrated in recent murine studies is a dual-action lipid nanoparticle (dual-LNP) co-loaded with VISTA-specific siRNA and a TLR9 agonist (CpG oligonucleotide) (103). This design ensures codelivery to tumor-infiltrating myeloid cells, achieving simultaneous silencing of the VISTA checkpoint and activation of innate immunity via TLR9 stimulation. The synergy between these cargoes was profound, rejecting large established tumors in melanoma (YUMM1.7, B16F10) and colon carcinoma (MC38) models where single-component LNPs failed, and outperformed soluble CpG plus a VISTA monoclonal antibody with reduced systemic inflammation (103). Other smart nanocarriers are designed for spatial control of release. A pH-responsive Pickering nanoemulsion utilized multi-sensitive nanogels to achieve specified spatial delivery: in the acidic TME, it disassembled to release an immune checkpoint inhibitor extracellularly while delivering a chemotherapeutic agent (doxorubicin) into tumor cells, inducing immunogenic cell death and a strong combined antitumor immune response (95). Similarly, in animal models, microneedle-based transcutaneous patches have been engineered to locally deliver nanocapsules loaded with anti-PD-1 antibodies, incorporating an IDO inhibitor (1-MT) into the carrier material itself, facilitating sustained release and enhanced retention in the melanoma TME (89).

The concept of local or regional administration of immunotherapies is gaining significant traction for skin cancers, given their accessibility, with the goal of maximizing intratumoral drug concentration while minimizing systemic exposure and toxicity (104, 105). A compelling study demonstrated that peritumoral (local) administration of a triple antibody combination targeting PD-1, 4-1BB (CD137), and VISTA into mouse ear skin tumors led to complete tumor elimination at a lower dose than required intravenously, without inducing measurable hepatic toxicity (104). This local treatment not only cleared the primary tumor but also induced systemic immunity, causing regression of untreated distal tumors and establishing long-term immune memory (104) (**Figure 4**). Similar principles apply to intratumoral delivery of other immunomodulators. Intratumoral administration of a STING-activating nanovaccine was more effective than subcutaneous injection in promoting antigen-specific T cell infiltration and tumor control, a finding relevant for designing VISTA-targeted nanomedicine administration routes (106). Beyond liquid injections, sustained-release depots offer another local strategy. Biodegradable polymeric microparticles loaded with immunomodulatory antibodies (e.g., anti-CTLA-4) can provide localized, sustained release over weeks, showing therapeutic efficacy comparable to conventional emulsions but with lower systemic antibody levels and reduced risk of adverse effects (107).

**Figure 4:**
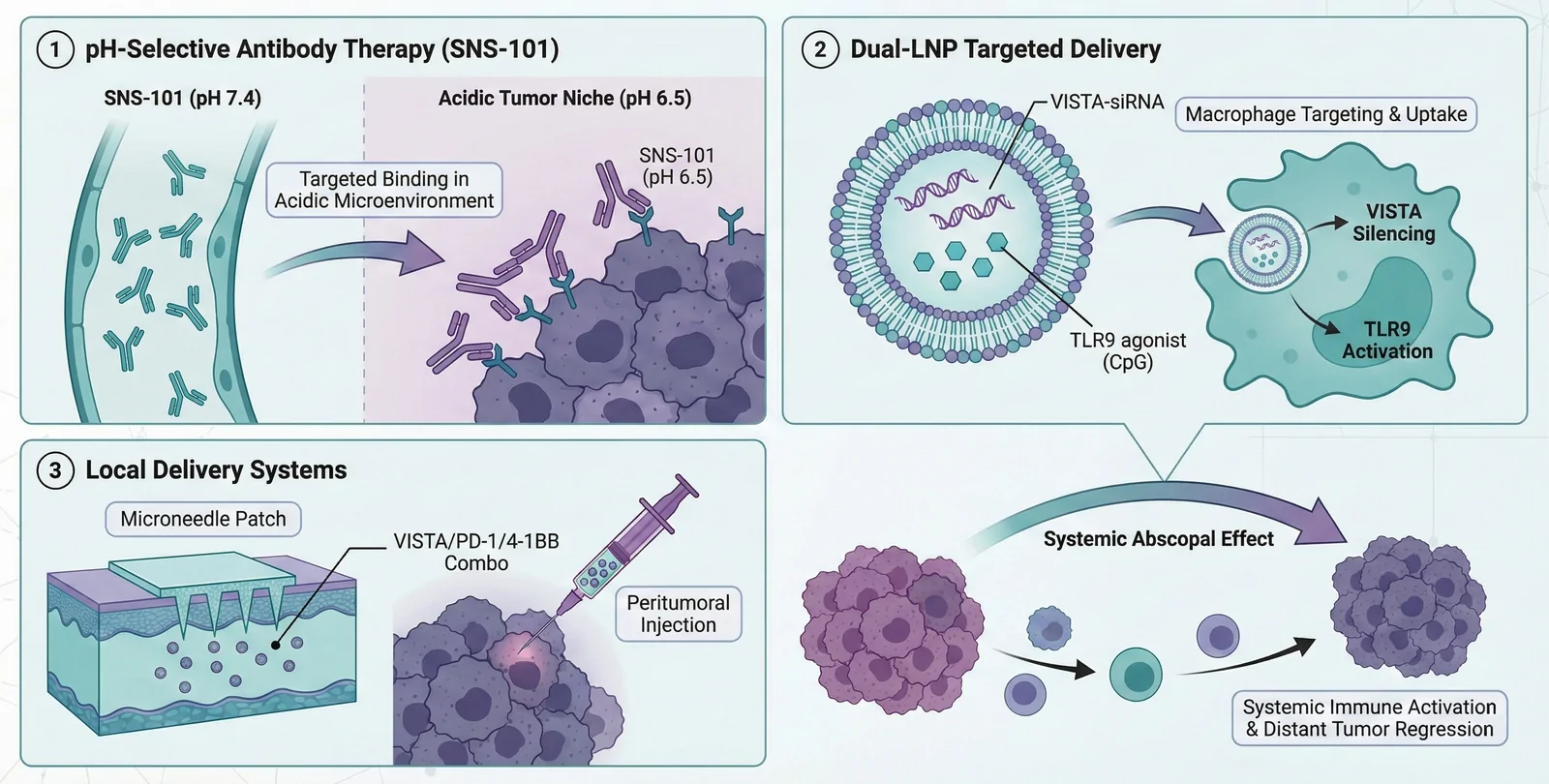
Next-generation therapeutic approaches targeting the VISTA axis in skin cancer.

Given the interconnectivity of immunosuppressive pathways, rational combination therapies are paramount. Preclinical evidence strongly supports combining VISTA blockade with other immunotherapies. VISTA antibodies synergize with anti-PD-1, anti-CTLA-4, or agonists like anti-4-1BB (29, 98, 99, 104). The development of bispecific antibodies represents an elegant method to co-target pathways. While not yet reported for VISTA specifically, bispecifics targeting PD-L1/LAG-3 (IBI323) or LAG-3/PD-L1 (ABL501) demonstrate superior immune stimulation and antitumor efficacy compared to parent antibody combinations, partly through novel functions like bridging immune cells (79, 108). This format could be adapted for VISTA in combination with PD-1 or other checkpoints. Furthermore, combinations extending beyond T-cell checkpoints are logical. Since the acidic, adenosine-rich TME can upregulate VISTA and other checkpoints, combining VISTA inhibition with adenosine pathway blockade (e.g., A2AR antagonists like CPI-444 or anti-CD73 antibodies like MEDI9447) is a promising strategy (81, 109). This is supported by findings that exosomal CD73 contributes to anti-PD-1 resistance in melanoma (110). Combinations with conventional therapies that modulate the TME are also effective. Chemotherapy agents like paclitaxel or oxaliplatin can induce immunogenic cell death (ICD) but may also upregulate checkpoints like PD-L1 and CD47; combining them with checkpoint blockade (including potential VISTA targeting) enhances efficacy (111, 112). Similarly, repurposed drugs like metformin or disulfiram can alter checkpoint expression (e.g., promoting PD-L1 degradation or upregulation) and improve responses to immunotherapy, suggesting potential synergy with VISTA-targeted agents (38, 113, 114).

Despite the promise, several challenges must be navigated for successful clinical translation of VISTA-directed therapies. Patient stratification is crucial, as not all tumors rely equally on the VISTA pathway. Biomarkers such as high VISTA expression on tumor-infiltrating myeloid cells or lymphocytes, particularly in post-PD-1 therapy biopsies, may identify patients most likely to benefit (96). Managing potential toxicities remains a focus; pH-selective antibodies and local delivery platforms are specifically designed to improve the therapeutic window by limiting on-target, off-tumor effects (29, 104). Optimizing local delivery systems for ease of clinical application and ensuring they induce robust systemic abscopal effects are ongoing areas of development (104, 106). Furthermore, the choice of optimal combination partners will require a deeper understanding of the dominant resistance mechanisms in individual patients. Finally, integrating VISTA-targeting strategies into the broader framework of precision immuno-oncology, potentially alongside tumor-targeted 4-1BB agonists, metabolic modulators, or novel scaffolds with innate immune adjuvanticity, will be key to building the next generation of skin cancer immunotherapies (101, 115, 116). The journey from mechanistic understanding to clinical implementation for VISTA is well underway. Although large-scale human efficacy data are still pending, the existing preclinical framework suggests that VISTA blockade could offer a unique and potent tool to reinvigorate antitumor immunity in cutaneous malignancies refractory to current standards of care.

## 6 Preclinical and clinical evidence for VISTA-targeted therapy in melanoma and cSCC

The clinical investigation of VISTA-targeted therapies in skin cancers is nascent, yet a growing body of preclinical evidence and correlative clinical data strongly supports its therapeutic potential, particularly in contexts of resistance to existing immune checkpoint inhibitors. In cutaneous melanoma, the expression landscape of VISTA is complex and varies across subtypes. A single-cell RNA sequencing study of acral melanoma revealed a generally suppressed immune microenvironment compared to nonacral cutaneous melanoma, characterized by lower effector T and NK cell infiltration. Notably, VISTA was expressed in a substantial 58.3% of myeloid cells within these tumors, identifying it as a prominent checkpoint alongside PD-1, LAG-3, and TIGIT in this challenging subtype (42). In treatment-naïve melanoma patients, VISTA was found among the most abundant checkpoint receptors, though its expression, along with TIM-3, was predominantly localized to non-T cells (48). This pattern underscores its role in modulating the myeloid compartment, a key consideration for therapeutic targeting. The prognostic significance of VISTA appears to be context-dependent. In uveal melanoma (UM), a subtype notorious for its resistance to conventional anti-PD-1/CTLA-4 therapy, VISTA expression has been linked to tumor aggressiveness and shorter overall survival (45). Further characterization in a French UM cohort confirmed VISTA expression on tumor-infiltrating lymphocytes, with higher levels associated with better prognosis in certain contexts, highlighting the need for detailed spatial and cellular resolution in biomarker analysis (46). This contrasts with findings in other cancers like craniopharyngioma, where high VISTA expression correlated with better survival, and in endometrial cancer, where VISTA on immune cells was an independent predictor of improved outcome (47, 117).

In cSCC, which now sees anti-PD-1 therapy as a standard of care for advanced disease (118), VISTA represents a compelling next-generation target. Multiplexed analysis of primary cSCC tissues demonstrated that VISTA is expressed in 48% of tumors. Critically, high VISTA expression correlated more strongly than PD-L1 with greater T-cell infiltration and activation markers like Ki-67 and granzyme B (44). This finding is pivotal as it suggests that VISTA is not merely a marker of an inert, immune-excluded “cold” tumor, but may be upregulated as part of an adaptive resistance mechanism in immunologically “hot” but still progressing tumors. This pattern mirrors the established adaptive resistance mechanism for PD-L1, where its expression is induced by IFN-γ from tumor-infiltrating lymphocytes (58).

While clinical confirmation is pending, preclinical models robustly validate VISTA as a promising therapeutic target in skin cancer. The inaugural landmark study defining the ‘causal’ role of VISTA in skin cancer was conducted by Le Mercier et al., who demonstrated that targeted VISTA blockade significantly enhanced antitumor immunity and suppressed tumor growth in murine models of melanoma (119). As highlighted in our discussion of drug delivery systems, advanced platforms like the Dual-LNP (co-delivering VISTA-siRNA and a TLR9 agonist) robustly validate VISTA as a therapeutic target in murine melanoma models, demonstrating the capacity to safely reprogram the immunosuppressive myeloid compartment and reject established tumors (103). This nanotechnology approach directly addresses the challenge of selectively modulating the immunosuppressive myeloid compartment within the tumor. Similarly, the success of local, peritumoral administration of a triple combination (targeting PD-1, 4-1BB, and VISTA) provides a powerful preclinical proof-of-concept. By eradicating primary tumors and inducing systemic protective memory without hepatic toxicity, it underscores the potential of localized checkpoint blockade to maximize antitumor efficacy while minimizing systemic irAEs (104). This study provides a powerful preclinical proof-of-concept for localized, combination checkpoint blockade incorporating VISTA as a means to achieve potent antitumor effects while minimizing systemic irAEs.

The role of VISTA in the context of other immunotherapies is also being elucidated. Research in chimeric antigen receptor T (CAR-T) cell therapy models for B-cell malignancies and melanoma revealed that VISTA expression on endogenous T cells is regulated by IFN-γ signaling from the CAR-T products. In settings where this IFN-γ signal was absent, VISTA levels remained elevated, leading to attenuated cytotoxicity of endogenous T cells and reduced survival. Blocking VISTA signaling enhanced dendritic cell-induced proliferation and cytokine production by syngeneic T cells, suggesting that VISTA inhibition could be a strategy to bolster endogenous immune responses and prevent relapse following CAR-T therapy (120). This interplay between VISTA and IFN-γ pathways connects directly to known mechanisms of acquired resistance to PD-1 blockade. Resistance to anti-PD-1 in melanoma and lung cancer has been associated with defects in IFN-receptor signaling (e.g., JAK1/2 mutations) or antigen presentation (e.g., B2M loss) (84, 121). In such resistant tumors, where IFN-γ-mediated induction of other checkpoints like PD-L1 may be impaired, VISTA—which can be regulated independently or through alternative pathways—may emerge as a dominant suppressor. Furthermore, in melanoma, tumor expression of the immune checkpoint ligand CD155 has been associated with resistance to anti-PD-1, correlating with a higher fraction of PD-1+CD8+ T cells within the tumor (122). This suggests that multiple non-redundant inhibitory pathways, potentially including VISTA, can converge to maintain T-cell dysfunction even after PD-1 blockade.

While immune checkpoint inhibitors targeting PD-1 and CTLA-4 have achieved established clinical success and represent the current standard of care, clinical evidence specifically for VISTA-targeted monotherapy in skin cancers remains in its infancy and is still awaited from ongoing early-phase trials. Consequently, indirect clinical data and experiences with established immunotherapies must be used to infer its theoretical potential. For instance, the efficacy of anti-PD-1 in cSCC, demonstrated in trials like KEYNOTE-629 with an objective response rate of 34.3% (118), establishes that these tumors are immunologically responsive. The finding that VISTA is highly expressed in human cSCC tissues with active but suppressed T-cell infiltrates directly points to a population that may hypothetically benefit from VISTA blockade, particularly after progression on anti-PD-1 (44). Similarly, while nivolumab and ipilimumab have revolutionized melanoma treatment (8), a significant proportion of patients still do not respond or relapse. The identification of an innate anti-PD-1 resistance (IPRES) signature, enriched in mesenchymal and wound-healing pathways (69), suggests that overcoming resistance may require targeting alternative immunosuppressive axes beyond the PD-1/CTLA-4 circuit. The prominent expression of VISTA on myeloid cells, which are key players in such pro-tumorigenic microenvironments (123), positions VISTA inhibitors as rational partners in combination regimens.

Early-phase trials exploring other novel immune agonists provide a framework for how VISTA-targeted agents might be developed. Studies of glucocorticoid-induced TNFR-related protein (GITR) or 4-1BB agonists in combination with PD-1 blockade have shown manageable safety profiles and some clinical activity, though often modest as monotherapies (91, 124). These experiences highlight the importance of patient selection and combination strategies. In this vein, retrospective analyses hint at potential synergies. For example, a retrospective study in metastatic melanoma patients suggested that those receiving metformin concurrently with immune checkpoint inhibitors (ipilimumab and/or anti-PD-1) had favorable trends in response rates and survival, though not statistically significant in this small cohort (125). Preclinical studies indicate metformin can inhibit melanoma invasion and metastasis (126) and enhance antitumor immunity (127). While not directly involving VISTA, such research underscores the broader principle that modulating the TME can enhance checkpoint immunotherapy, a principle directly applicable to VISTA’s role in myeloid cell function. To provide a clear distinction between agents currently undergoing human evaluation and those exhibiting early experimental promise, **Table 2** summarizes the key VISTA-targeted therapeutics.

**Table 2:** Summary of key VISTA-targeted therapeutic agents in clinical and preclinical development.

Agent/strategy	Class & mechanism of action	Therapy type	Development phase	Target malignancies/models
SNS-101	Fully human IgG1 mAb (pH-selective VISTA antagonist)	Monotherapy & Combination (with anti-PD-1)	Phase 1/2 Clinical (NCT05864144)	Advanced Solid Tumors (incl. Melanoma, cSCC, Colorectal, H&N)
KVA12123	Fully human IgG1-kappa mAb (VISTA antagonist; blocks its interactions)	Monotherapy & Combination (with anti-PD-1)	Phase 1/2 Clinical (NCT05708950)	Advanced Solid Tumors (incl. Melanoma, NSCLC)
Triple-Antibody Combo (anti-VISTA, anti-PD-1, anti-4-1BB)	Monoclonal Antibodies (Combination of checkpoint inhibitors & co-stimulatory agonist)	Combination Therapy	Preclinical (*In vivo*)	Skin Cancer (Syngeneic murine ear tumor models)
Dual-LNP (VISTA-siRNA + CpG)	Lipid Nanoparticle (Gene silencing + TLR9 activation)	Combination Therapy (siRNA + Agonist)	Preclinical (*In vivo*)	Melanoma (B16F10, YUMM1.7), Colon Carcinoma (MC38)
SG7	Monoclonal Antibody (pan-species VISTA antagonist)	Monotherapy & Combination (with anti-PD-1)	Preclinical (*In vivo*)	Multiple Syngeneic Murine Models
L557-0155	Small Molecule (Inhibitor of VSIG-8/VISTA interaction)	Monotherapy	Preclinical (*In vivo*)	Melanoma (Murine Models)
M351-0056	Small Molecule (VISTA ligand, modulates JAK2-STAT2 pathway)	Monotherapy	Preclinical (*In vivo/In vitro*)	Psoriasis-like dermatitis/Inflammation Models

NSCLC, non-small cell lung cancer; mAb, monoclonal antibody; LNP, lipid nanoparticle.

In conclusion, a convergence of preclinical and emerging clinical evidence solidifies VISTA as a promising immunotherapeutic target in melanoma and cSCC. Its expression in key immunosuppressive myeloid populations, its correlation with T-cell-inflamed but non-responding TME, and its potential role in resistance to existing therapies provide a strong biological rationale. Preclinical studies using advanced delivery systems like nanoparticles or local peritumoral injection demonstrate potent antitumor efficacy and inform novel clinical development strategies aimed at maximizing therapeutic index. The ongoing challenge is to translate these insights into successful clinical trials, which will require careful patient stratification based on VISTA expression patterns, strategic combination with other immunomodulators, and potentially innovative routes of administration to safely unlock the full potential of VISTA blockade in overcoming immune resistance in skin cancers.

## 7 Limitations, challenges, and future directions for VISTA-directed immunotherapy

Despite the compelling biological rationale for VISTA-directed therapies, it is crucial to explicitly acknowledge the limitations of the current evidence base. First, a substantial portion of the detailed mechanistic understanding of VISTA’s receptor-ligand interactions, metabolic regulation, and prognostic value is currently extrapolated from other malignancies (such as lung, breast, and hematological cancers) or non-cancer autoimmune models. While these pan-cancer and cross-disciplinary insights provide a vital and highly plausible theoretical framework for cutaneous oncology, skin cancers possess distinct mutational landscapes (e.g., high UV-induced TMB) and unique neuroectodermal or epithelial microenvironments. Therefore, a critical future direction is the rigorous, tissue-specific validation of these extrapolated mechanisms exclusively within primary and metastatic melanoma and cSCC. Second, there is an overreliance on preclinical data to project therapeutic success. Although advanced delivery systems and multi-target regimens exhibit robust efficacy *in vivo*, their translational potential remains speculative pending human trials. This is compounded by the inherent limitations of syngeneic murine models, which fail to fully recapitulate the uniquely high tumor mutational burden (TMB) and complex stromal evolution characteristic of human skin cancers. Third, the contradictory findings regarding VISTA expression and prognosis pose a major translational hurdle. VISTA may signify a deeply suppressed ‘cold’ tumor in bulk tissue analyses, yet represent a reactive, ‘adaptive resistance’ response when expressed on specific TIL subsets. This biological paradox is further exacerbated by a lack of standardized diagnostic assays, validated antibodies, and consensus scoring cut-offs, hindering cross-trial comparisons. Finally, the safety of proposed combination treatment regimens remains highly uncertain. While dual or triple checkpoint blockade conceptually maximizes T-cell reinvigoration, the cumulative risk of overlapping, severe irAEs in human patients is currently unknown, underscoring the critical need for localized delivery innovations before such aggressive systemic regimens can be safely adopted in the clinic.

Translating the compelling preclinical rationale for VISTA blockade into effective and safe clinical regimens for skin cancer presents distinct challenges that stem from its unique biology and the complexity of the tumor immune microenvironment. A primary hurdle is the multifaceted and context-dependent nature of VISTA signaling, which functions as both a ligand on antigen-presenting cells and a receptor on T cells, engaging binding partners like LRIG1 to suppress T cell receptor signaling (34, 120). This complexity is compounded by its frequent co-expression with other inhibitory pathways. Although direct evidence in skin cancer is still emerging, observations from other solid tumors are instructive. In squamous cell lung carcinoma, for instance, a subset of patients exhibits a coordinated upregulation of multiple checkpoints, including VISTA, PD-1, CTLA-4, LAG-3, and TIGIT, defining an “exhausted immune class” characterized by a T-cell-inflamed yet profoundly immunosuppressive microenvironment that is resistant to single-agent checkpoint blockade (128). This pattern of co-expression and potential functional redundancy is a common mechanism of adaptive resistance, as evidenced by studies showing that blockade of one checkpoint like PD-1 can lead to the compensatory upregulation of others, such as TIM-3 or VISTA, to maintain immune suppression (73, 96). Therefore, a fundamental challenge is determining whether VISTA functions as a dominant, parallel, or compensatory resistance pathway in a given patient, which directly informs rational combination strategies.

Clinical development of VISTA-targeted agents must also navigate the significant toxicity associated with systemic immune checkpoint inhibition, particularly with combinatorial approaches. The improved efficacy of combined anti-CTLA-4 and anti-PD-1 in melanoma comes at the cost of high-grade adverse events in a majority of patients (8, 71). Adding a third systemic agent targeting VISTA could exacerbate these toxicities, limiting its therapeutic window. This concern underscores the critical need for innovative delivery strategies to enhance the therapeutic index. Preclinical models strongly support localized administration, such as peritumoral or intratumoral delivery, which can achieve potent antitumor effects and induce systemic immunity while minimizing systemic exposure and associated toxicity (104, 106). Furthermore, advanced drug delivery systems like nanoparticles offer the potential for tumor-selective accumulation and controlled release. For example, a dual-action lipid nanoparticle co-delivering VISTA-specific siRNA and a TLR9 agonist synergistically rejected established tumors with reduced systemic cytokine production compared to soluble antibody therapy (103). Similarly, pH-responsive nanocarriers designed for spatially controlled delivery of immunomodulators and chemotherapeutic agents to different tumor compartments represent a promising platform to enhance combination efficacy while mitigating off-target effects (95). The future clinical testing of VISTA inhibitors must prioritize these localized or targeted delivery approaches to separate efficacy from toxicity.

Identifying robust predictive biomarkers is paramount for the successful clinical development of VISTA-directed therapies and for patient stratification. Given VISTA’s role in resistance, its expression patterns may serve as a dynamic biomarker. In metastatic melanoma patients who developed acquired resistance to anti-PD-1 therapy, a significant increase in the density of VISTA-positive tumor-infiltrating lymphocytes was a common finding, suggesting VISTA upregulation as a mechanism of adaptive escape (96). Thus, assessing VISTA expression on immune cells within the TME, particularly on myeloid subsets and T cells, before and during treatment may identify patients likely to benefit from or develop resistance to VISTA blockade. Beyond protein expression, transcriptional profiling offers a broader view. The aforementioned “exhausted immune class” signature, which includes VISTA, identifies tumors with a specific immunosuppressive biology that may be particularly amenable to multi-checkpoint inhibition (128). Computational frameworks like Tumor Immune Dysfunction and Exclusion (TIDE), which model dysfunction in T-cell-inflamed tumors and exclusion in non-inflamed tumors, can integrate VISTA-related signatures with other features to predict response to immunotherapy (129). Furthermore, baseline features associated with response to existing immunotherapies, such as high TMB and an interferon-gamma (IFN-γ) gene signature, also correlate with pathologic response to neoadjuvant combination checkpoint blockade in melanoma and may help enrich for populations where adding VISTA inhibition could be most effective (130). Conversely, elevated serum levels of immunosuppressive metabolites like kynurenine, a product of IDO activity, have been linked to worse survival on anti-PD-1 therapy, highlighting metabolic pathways as potential biomarkers and co-targets (131). A multi-modal biomarker approach incorporating VISTA protein expression, genomic and transcriptomic signatures, and soluble factors will be essential to guide therapy.

The future therapeutic landscape for VISTA will likely be dominated by rational combination strategies designed to overcome specific resistance mechanisms within the TME. One clear direction is combination with existing PD-1/PD-L1 axis inhibitors, based on strong preclinical synergy and clinical evidence of VISTA upregulation post-PD-1 blockade (96, 103). However, given the heterogeneity of resistance, other combinations are equally compelling. Targeting myeloid cells, a primary site of VISTA expression and function, is a logical strategy. Combining VISTA blockade with agents that activate myeloid cells, such as TLR agonists (as in the dual-LNP platform), CD40 agonists, or STING agonists, could convert immunosuppressive myeloid populations into immunostimulatory ones, breaking a key barrier to T-cell function (103, 132). Similarly, combining VISTA inhibition with therapies that target other non-redundant immunosuppressive pathways in the TME, such as adenosine signaling (via A2AR antagonists or CD73 inhibitors) or TGF-β, holds significant promise (56, 81, 82). These pathways contribute to T-cell exclusion and dysfunction independently of the PD-1/VISTA axis, and their blockade could be synergistic (82). Emerging data also suggest that modulating host metabolism with agents like metformin can enhance the efficacy of checkpoint inhibitors through pleiotropic immunomodulatory effects, presenting another viable combination partner for VISTA-targeted therapy (38).

Finally, the next generation of VISTA-directed therapies will extend beyond simple antibody blockade. The discovery of LRIG1 as a key binding partner opens the door to targeting this specific interaction (34). The pH-sensitive nature of VISTA’s suppressive function offers a unique opportunity for engineering conditional inhibitors or activators that are selectively active in the acidic TME, potentially increasing specificity. Nanomedicine platforms will continue to evolve, enabling not just co-delivery with other immunomodulators but also with conventional therapies like chemotherapy or radiotherapy that can induce immunogenic cell death and alter the TME to be more receptive to checkpoint blockade (95, 133). Furthermore, integrating VISTA blockade with novel co-stimulatory approaches, such as agonists of 4-1BB (CD137), GITR, or CD27, could provide the necessary signal to revitalize exhausted T cells whose function is being restrained by VISTA (91, 104, 134). For skin cancers amenable to local intervention, strategies like microneedle-based transcutaneous delivery or injectable hydrogels could sustain local release of VISTA inhibitors in combination with other agents, maximizing immune activation at the tumor site while minimizing systemic dispersion (89). The path forward requires a nuanced understanding of VISTA biology within the ecosystem of each patient’s tumor, leveraging advanced biomarkers to design personalized combination regimens delivered through innovative platforms that maximize antitumor immunity and safety.

## 8 Conclusions and perspectives

The journey of cancer immunotherapy, from a promising concept to a cornerstone of modern oncology, underscores the power of harnessing the host immune system (15, 135). In skin cancers, particularly melanoma and cSCC, ICB targeting CTLA-4 and PD-1/PD-L1 has revolutionized treatment paradigms, delivering durable responses previously deemed unattainable (8, 9, 136). However, the reality remains that a substantial proportion of patients derive no benefit or experience relapse, highlighting the complex and heterogeneous nature of immune evasion (11, 18, 70). This therapeutic plateau necessitates the exploration of novel targets and the development of more sophisticated, integrated approaches. Within this landscape, VISTA emerges not merely as another inhibitory molecule to blockade, but as a unique and pivotal component whose biology offers distinct opportunities to refine and advance the next generation of skin cancer immunotherapies (10, 17).

The future of VISTA-directed therapy lies in its strategic integration into multimodal regimens that address the multifaceted immunosuppressive TME. The success of the anti-lymphocyte-activation gene 3 (LAG-3) antibody relatlimab in combination with nivolumab for metastatic melanoma validated the principle that targeting co-inhibitory pathways beyond PD-1 and CTLA-4 can yield superior efficacy with a manageable toxicity profile, establishing a blueprint for future combination strategies (12). VISTA is poised to follow a similar trajectory. Its expression on both myeloid and T cell subsets within the skin cancer TME positions it as a key regulator of a broader immunosuppressive network (21, 103). Therefore, rational combinations are paramount. The synergy observed in preclinical models between VISTA blockade and PD-1 inhibition, or with co-stimulatory agonists like anti-4-1BB, provides a strong mechanistic foundation (75, 104). These combinations aim to simultaneously remove multiple layers of inhibition while providing positive signals to rejuvenate exhausted T cells, effectively altering the “cancer-immune set point” towards a more permissive state for antitumor immunity (6, 11).

A critical and distinguishing feature of VISTA is its pH-sensitive function, which presents a unique therapeutic design opportunity. As discussed, this unique localization of its active immunosuppressive form within the acidic tumor niche presents a strategic therapeutic design opportunity (103). Future drug development can leverage this biology to engineer conditional therapies—such as antibodies or small molecules with enhanced binding at low pH—that preferentially disrupt VISTA-mediated suppression in the TME while sparing its potential physiological roles in peripheral tissues. This approach aligns with the broader goal of enhancing therapeutic specificity to mitigate irAEs, a significant challenge with current systemic checkpoint inhibitors (8, 21). Furthermore, if successfully translated from bench to bedside, innovative drug delivery systems (DDS) will be instrumental in realizing the full potential of VISTA-targeted combinations. Nanoparticle platforms capable of co-delivering VISTA-silencing siRNA with immune stimulators like TLR agonists have demonstrated remarkable preclinical efficacy, ensuring both agents reach the same immunosuppressive myeloid cells to synergistically reprogram the TME (101, 103). Such nanomedicine strategies not only improve targeting and retention but also allow for controlled release, potentially overcoming issues of poor biodistribution and reducing systemic cytokine storms associated with soluble agonists (103, 106).

For skin cancers, the accessibility of the tumor site offers a particularly advantageous route for intervention. Local or peritumoral delivery of immunomodulatory antibodies, including those targeting VISTA, PD-1, and 4-1BB, has shown compelling preclinical results (104). This approach achieves potent local and systemic antitumor immunity with significant dose-sparing and a markedly reduced risk of off-target hepatic toxicity compared to intravenous administration (104). It represents a paradigm shift for managing locally advanced or multiple primary cSCCs and melanomas, especially in cases where surgery is challenging or for patients who are poor candidates for systemic immunotherapy due to comorbidities (1, 137). Integrating VISTA blockade into such localized regimens, potentially via injectable hydrogels or dissolvable microneedle patches, could maximize immune activation at the tumor site while minimizing systemic exposure, offering a safer and more effective treatment modality for early-stage or locally advanced disease (101, 104).

Looking ahead, several frontiers must be charted to successfully integrate VISTA into clinical practice. First, a deeper understanding of VISTA’s interaction with other immune pathways in the dynamic process of cancer immunoediting is essential (13). Research must delineate its role in primary resistance to existing therapies and in acquired resistance following initial response (70). Second, the development of robust predictive biomarkers is a non-negotiable prerequisite. Given the heterogeneity of VISTA expression across patients and tumor types, identifying which patients with melanoma or cSCC are most likely to benefit from VISTA-directed therapy is crucial (4, 136). Biomarkers may include levels of VISTA expression on specific immune subsets, the pH profile of the TME, or genetic signatures associated with VISTA-mediated suppression. Third, the therapeutic scope should expand. While most research focuses on advanced disease, the potential of VISTA blockade in the neoadjuvant setting for high-risk skin cancers warrants exploration, with the aim of reducing tumor burden and eradicating micrometastatic disease prior to surgery (137). Additionally, its role in rare or aggressive subtypes like acral lentiginous melanoma or Merkel cell carcinoma, which may harbor distinct immune microenvironments, needs investigation (3, 137).

Finally, the vision for the next generation extends beyond checkpoint blockade alone. The convergence of VISTA-targeted strategies with other burgeoning modalities—such as cancer vaccines, adoptive cell therapies (CAR-T), oncolytic viruses, and metabolic modulators—will define the future of personalized immuno-oncology (5, 106, 127, 138). For instance, combining VISTA blockade with a personalized neoantigen vaccine could simultaneously provide tumor-specific T cell targets and remove a key barrier to their effector function. Similarly, modulating the TME with metformin to improve T cell fitness, alongside VISTA inhibition, could be a potent strategy to reverse T cell exhaustion (127). The overarching principle is to move from a “one-size-fits-all” blockade to a “battlespace” vision that employs a coordinated array of therapeutic tools to overcome the diverse and adaptive resistance mechanisms employed by tumors (18).

In conclusion, VISTA represents a compelling and biologically rational target whose integration into the therapeutic arsenal against skin cancer is poised to address the limitations of current immunotherapies. Its unique pH-dependent mechanism, dual cellular expression, and synergistic potential with existing and emerging modalities position it at the forefront of next-generation strategies. The path forward requires a concerted effort combining fundamental biological research, innovative bioengineering for targeted delivery, and smart clinical trial design guided by predictive biomarkers. By embracing this integrated approach, the field can advance towards the ultimate goal of durable, and potentially curative, immune-mediated control for all patients with skin cancer.

## References

[1] Burns C Kubicki S Nguyen Q Aboul-Fettouh N Wilmas K Chen O . Advances in cutaneous squamous cell carcinoma management. Cancers Basel. (2022) 14. doi: 10.3390/cancers14153653 35954316 PMC9367549

[2] Zhang W Zeng W Jiang A He Z Shen X Dong X . Global, regional and national incidence, mortality and disability-adjusted life-years of skin cancers and trend analysis from 1990 to 2019: An analysis of the Global Burden of Disease Study 2019. Cancer Med. (2021) 10:4905–22. doi: 10.1002/cam4.4046 34105887 PMC8290243

[3] Bradford P Goldstein A McMaster M Tucker M . Acral lentiginous melanoma: incidence and survival patterns in the United States, 1986-2005. Arch Dermatol. (2009) 145:427–34. doi: 10.1001/archdermatol.2008.609 PMC2735055 19380664

[4] Thomas R Al-Khadairi G Decock J . Immune checkpoint inhibitors in triple negative breast cancer treatment: promising future prospects. Front Oncol. (2020) 10:600573. doi: 10.3389/fonc.2020.600573 33718107 PMC7947906

[5] Mishra A Ali A Dutta S Banday S Malonia S . Emerging trends in immunotherapy for cancer. Diseases. (2022) 10. doi: 10.3390/diseases10030060 36135216 PMC9498256

[6] Topalian S Drake C Pardoll D . Immune checkpoint blockade: a common denominator approach to cancer therapy. Cancer Cell. (2015) 27:450–61. doi: 10.1016/j.ccell.2015.03.001 25858804 PMC4400238

[7] Wei S Duffy C Allison J . Fundamental mechanisms of immune checkpoint blockade therapy. Cancer Discov. (2018) 8:1069–86. doi: 10.1158/2159-8290.cd-18-0367 30115704

[8] Hodi F Chiarion-Sileni V Gonzalez R Grob J Rutkowski P Cowey C . Nivolumab plus ipilimumab or nivolumab alone versus ipilimumab alone in advanced melanoma (CheckMate 067): 4-year outcomes of a multicentre, randomised, phase 3 trial. Lancet Oncol. (2018) 19:1480–92. doi: 10.1016/s1470-2045(18)30700-9 30361170

[9] Seth R Messersmith H Kaur V Kirkwood J KudChadkar R McQuade J . Systemic therapy for melanoma: ASCO guideline. J Clin Oncol. (2020) 38:3947–70. doi: 10.1200/jco.20.00198 32228358

[10] Ziogas D Theocharopoulos C Lialios P Foteinou D Koumprentziotis I Xynos G . Beyond CTLA-4 and PD-1 inhibition: novel immune checkpoint molecules for melanoma treatment. Cancers Basel. (2023) 15. doi: 10.3390/cancers15102718 37345056 PMC10216291

[11] Chen D Mellman I . Elements of cancer immunity and the cancer-immune set point. Nature. (2017) 541:321–30. doi: 10.1038/nature21349 28102259

[12] Kreidieh F Tawbi H . The introduction of LAG-3 checkpoint blockade in melanoma: immunotherapy landscape beyond PD-1 and CTLA-4 inhibition. Ther Adv Med Oncol. (2023) 15:17588359231186027. doi: 10.1177/17588359231186027 37484526 PMC10357068

[13] Schreiber R Old L Smyth M . Cancer immunoediting: integrating immunity’s roles in cancer suppression and promotion. Science. (2011) 331:1565–70. doi: 10.1126/science.1203486 21436444

[14] Arina A Corrales L Bronte V . Enhancing T cell therapy by overcoming the immunosuppressive tumor microenvironment. Semin Immunol. (2016) 28:54–63. doi: 10.1016/j.smim.2016.01.002 26872631

[15] Palucka A Coussens L . The basis of oncoimmunology. Cell. (2016) 164:1233–47. doi: 10.1016/j.cell.2016.01.049 26967289 PMC4788788

[16] Muntyanu A Netchiporouk E Gerstein W Gniadecki R Litvinov I . Cutaneous immune-related adverse events (irAEs) to immune checkpoint inhibitors: a dermatology perspective on management. J Cutan Med Surg. (2021) 25:59–76. doi: 10.1177/1203475420943260 32746624

[17] Burugu S Dancsok A Nielsen T . Emerging targets in cancer immunotherapy. Semin Cancer Biol. (2018) 52:39–52. doi: 10.1016/j.semcancer.2017.10.001 28987965

[18] Hanahan D . Rethinking the war on cancer. Lancet. (2014) 383:558–63. doi: 10.1016/s0140-6736(13)62226-6 24351321

[19] Roy D Gilmour C Patnaik S Wang LL . Combinatorial blockade for cancer immunotherapy: targeting emerging immune checkpoint receptors. Front Immunol. (2023) 14:1264327. doi: 10.3389/fimmu.2023.1264327 37928556 PMC10620683

[20] Xu W Hiếu T Malarkannan S Wang L . The structure, expression, and multifaceted role of immune-checkpoint protein VISTA as a critical regulator of anti-tumor immunity, autoimmunity, and inflammation. Cell Mol Immunol. (2018) 15:438–46. doi: 10.1038/cmi.2017.148 29375120 PMC6068175

[21] Mahdavi GA Sadat RM Sanaei M Davaran S Kesharwani P Sahebkar A . Immune checkpoint blockade in melanoma: advantages, shortcomings and emerging roles of the nanoparticles. Int Immunopharmacol. (2022) 113:109300. doi: 10.1016/j.intimp.2022.109300 36252486

[22] Huang X Zhang X Li E Zhang G Wang X Tang T . VISTA: an immune regulatory protein checking tumor and immune cells in cancer immunotherapy. J Hematol Oncol. (2020) 13:83. doi: 10.1186/s13045-020-00917-y 32600443 PMC7325042

[23] ElTanbouly M Croteau W Noelle R Lines J . VISTA: a novel immunotherapy target for normalizing innate and adaptive immunity. Semin Immunol. (2019) 42:101308. doi: 10.1016/j.smim.2019.101308 31604531 PMC7233310

[24] Wang L Rubinstein R Lines JL Wasiuk A Ahonen C Guo Y . VISTA, a novel mouse Ig superfamily ligand that negatively regulates T cell responses. J Exp Med. (2011) 208:577–92. doi: 10.1084/jem.20100619 21383057 PMC3058578

[25] ElTanbouly M Zhao Y Nowak E Li J Schaafsma E Le MI . VISTA is a checkpoint regulator for naïve T cell quiescence and peripheral tolerance. Science. (2020) 367. doi: 10.1126/science.aay0524 31949051 PMC7391053

[26] ElTanbouly M Schaafsma E Smits N Shah P Cheng C Burns C . VISTA re-programs macrophage biology through the combined regulation of tolerance and anti-inflammatory pathways. Front Immunol. (2020) 11:580187. doi: 10.3389/fimmu.2020.580187 33178206 PMC7593571

[27] Slater B Han X Chen L Xiong Y . Structural insight into T cell coinhibition by PD-1H (VISTA). Proc Natl Acad Sci U A. (2020) 117:1648–57. doi: 10.1073/pnas.1908711117 31919279 PMC6983362

[28] Mehta N Maddineni S Mathews I Andres PSR Huang P Cochran J . Structure and functional binding epitope of V-domain Ig suppressor of T cell activation. Cell Rep. (2019) 28:2509–2516.e5. doi: 10.1016/j.celrep.2019.07.073 31484064

[29] Thisted T Smith F Mukherjee A Kleschenko Y Feng F Jiang Z . VISTA checkpoint inhibition by pH-selective antibody SNS-101 with optimized safety and pharmacokinetic profiles enhances PD-1 response. Nat Commun. (2024) 15:2917. doi: 10.1038/s41467-024-47256-x 38575562 PMC10995192

[30] Mulati K Hamanishi J Matsumura N Chamoto K Mise N Abiko K . VISTA expressed in tumour cells regulates T cell function. Br J Cancer. (2019) 120:115–27. doi: 10.1038/s41416-018-0313-5 30382166 PMC6325144

[31] Chen W Qie C Hu X Wang L Jiang J Liu W . A small molecule inhibitor of VSIG-8 prevents its binding to VISTA. Invest New Drugs. (2022) 40:690–9. doi: 10.1007/s10637-022-01244-4 35404016

[32] Olbromski M Mrozowska M Piotrowska A Smolarz B Romanowicz H . The VISTA/VSIG3/PSGL-1 axis: crosstalk between immune effector cells and cancer cells in invasive ductal breast carcinoma. Cancer Immunol Immunother. (2024) 73:136. doi: 10.1007/s00262-024-03701-w 38833004 PMC11150347

[33] Johnston RJ Su LJ Pinckney J Critton D Boyer E Krishnakumar A . VISTA is an acidic pH-selective ligand for PSGL-1. Nature. (2019) 574:565–70. doi: 10.1038/s41586-019-1674-5 31645726

[34] Ta H Roy D Zhang K Alban T Juric I Dong J . LRIG1 engages ligand VISTA and impairs tumor-specific CD8(+) T cell responses. Sci Immunol. (2024) 9:eadi7418. doi: 10.1126/sciimmunol.adi7418 38758807 PMC11334715

[35] Yasinska I Meyer N Schlichtner S Hussain R Siligardi G Casely-Hayford M . Ligand-receptor interactions of Galectin-9 and VISTA suppress human T lymphocyte cytotoxic activity. Front Immunol. (2020) 11:580557. doi: 10.3389/fimmu.2020.580557 33329552 PMC7715031

[36] Alanazi F As SH Alhazzani K Al-Dhfyan A Alshammari M Alotaibi M . Metformin attenuates V-domain Ig suppressor of T-cell activation through the aryl hydrocarbon receptor pathway in melanoma: *in vivo* and *in vitro* studies. Saudi Pharm J. (2022) 30:138–49. doi: 10.1016/j.jsps.2021.12.014 35528855 PMC9072704

[37] Opitz C Litzenburger U Sahm F Ott M Tritschler I Trump S . An endogenous tumour-promoting ligand of the human aryl hydrocarbon receptor. Nature. (2011) 478:197–203. doi: 10.1038/nature10491 21976023

[38] Liu W Wang Y Luo J Liu M Luo Z . Pleiotropic effects of metformin on the antitumor efficiency of immune checkpoint inhibitors. Front Immunol. (2020) 11:586760. doi: 10.3389/fimmu.2020.586760 33603734 PMC7884468

[39] Chen Y Sun J Liu J Wei Y Wang X Fang H . Aldehyde dehydrogenase 2-mediated aldehyde metabolism promotes tumor immune evasion by regulating the NOD/VISTA axis. J Immunother Cancer. (2023) 11. doi: 10.1136/jitc-2023-007487 38088186 PMC10711917

[40] Wang L Le MI Putra J Chen W Liu J Schenk A . Disruption of the immune-checkpoint VISTA gene imparts a proinflammatory phenotype with predisposition to the development of autoimmunity. Proc Natl Acad Sci U A. (2014) 111:14846–51. doi: 10.1073/pnas.1407447111 25267631 PMC4205642

[41] Prodeus A Abdul-Wahid A Sparkes A Fischer N Cydzik M Chiang N . VISTA.COMP - an engineered checkpoint receptor agonist that potently suppresses T cell-mediated immune responses. JCI Insight. (2017) 2. doi: 10.1172/jci.insight.94308 28931757 PMC5621893

[42] Li J Smalley I Chen Z Wu J Phadke M Teer J . Single-cell characterization of the cellular landscape of acral melanoma identifies novel targets for immunotherapy. Clin Cancer Res. (2022) 28:2131–46. doi: 10.1158/1078-0432.ccr-21-3145 35247927 PMC9106889

[43] Rosenbaum S Knecht M Mollaee M Zhong Z Erkes D McCue P . FOXD3 regulates VISTA expression in melanoma. Cell Rep. (2020) 30:510–524.e6. doi: 10.1016/j.celrep.2019.12.036 31940493 PMC6995351

[44] Kidacki M Cho C Lopez-Giraldez F Huang B He J Gaule P . PD-1H (VISTA) expression in cutaneous squamous cell carcinoma is correlated with T-cell infiltration and activation. J Invest Dermatol. (2025) 145:2313–2324.e6. doi: 10.1016/j.jid.2025.01.030 39983979 PMC12353361

[45] Issam SN Marnissi F Lakhdar A Karkouri M ElBelhadji M Badou A . The immune checkpoint VISTA is associated with prognosis in patients with Malignant uveal melanoma. Front Immunol. (2023) 14:1225140. doi: 10.3389/fimmu.2023.1225140 37662962 PMC10471992

[46] Lamas N Lassalle S Martel A Nahon-Estève S Macocco A Zahaf K . Characterisation of the protein expression of the emerging immunotherapy targets VISTA, LAG-3 and PRAME in primary uveal melanoma: insights from a southern French patient cohort. Pathol (Phila). (2023) 55:929–44. doi: 10.1016/j.pathol.2023.08.003 37863710

[47] Zong L Mo S Sun Z Lu Z Yu S Chen J . Analysis of the immune checkpoint V-domain Ig-containing suppressor of T-cell activation (VISTA) in endometrial cancer. Mod Pathol. (2022) 35:266–73. doi: 10.1038/s41379-021-00901-y 34493823

[48] Edwards J Tasker A Pires da SI Quek C Batten M Ferguson A . Prevalence and cellular distribution of novel immune checkpoint targets across longitudinal specimens in treatment-naïve melanoma patients: implications for clinical trials. Clin Cancer Res. (2019) 25:3247–58. doi: 10.1158/1078-0432.ccr-18-4011 30777877

[49] Lai C August S Albibas A Behar R Cho S Polak M . OX40+ regulatory T cells in cutaneous squamous cell carcinoma suppress effector T-cell responses and associate with metastatic potential. Clin Cancer Res. (2016) 22:4236–48. doi: 10.1158/1078-0432.ccr-15-2614 27034329 PMC4987192

[50] Xu W Dong J Zheng Y Zhou J Yuan Y Ta HM . Immune-checkpoint protein VISTA regulates antitumor immunity by controlling myeloid cell–mediated inflammation and immunosuppression. Cancer Immunol Res. (2019) 7:1497–510. doi: 10.1158/2326-6066.cir-18-0489 31340983 PMC6726548

[51] Zhang K Zakeri A Alban T Dong J Ta HM Zalavadia AH . VISTA promotes the metabolism and differentiation of myeloid-derived suppressor cells by STAT3 and polyamine-dependent mechanisms. Cell Rep. (2024) 43:113661. doi: 10.1016/j.celrep.2023.113661 38175754 PMC10851928

[52] Bozorgmehr N Okoye I Mashhouri S Lu J Koleva P Walker J . CD71(+) erythroid cells suppress T-cell effector functions and predict immunotherapy outcomes in patients with virus-associated solid tumors. J Immunother Cancer. (2023) 11. doi: 10.1136/jitc-2022-006595 37236637 PMC10230995

[53] Mastelic-Gavillet B Navarro RB Décombaz L Wang H Ercolano G Ahmed R . Adenosine mediates functional and metabolic suppression of peripheral and tumor-infiltrating CD8(+) T cells. J Immunother Cancer. (2019) 7:257. doi: 10.1186/s40425-019-0719-5 31601268 PMC6788118

[54] Antonioli L Pacher P Vizi E Haskó G . CD39 and CD73 in immunity and inflammation. Trends Mol Med. (2013) 19:355–67. doi: 10.1016/j.molmed.2013.03.005 23601906 PMC3674206

[55] Ohta A Gorelik E Prasad S Ronchese F Lukashev D Wong M . A2A adenosine receptor protects tumors from antitumor T cells. Proc Natl Acad Sci U A. (2006) 103:13132–7. doi: 10.1073/pnas.0605251103 16916931 PMC1559765

[56] Iannone R Miele L Maiolino P Pinto A Morello S . Adenosine limits the therapeutic effectiveness of anti-CTLA4 mAb in a mouse melanoma model. Am J Cancer Res. (2014) 4:172–81. PMC3960454 24660106

[57] Umansky V Shevchenko I Bazhin A Utikal J . Extracellular adenosine metabolism in immune cells in melanoma. Cancer Immunol Immunother. (2014) 63:1073–80. doi: 10.1007/s00262-014-1553-8 24756420 PMC11029545

[58] Taube J Anders R Young G Xu H Sharma R McMiller T . Colocalization of inflammatory response with B7-h1 expression in human melanocytic lesions supports an adaptive resistance mechanism of immune escape. Sci Transl Med. (2012) 4:127ra37. doi: 10.1126/scitranslmed.3003689 22461641 PMC3568523

[59] Fourcade J Sun Z Benallaoua M Guillaume P Luescher I Sander C . Upregulation of Tim-3 and PD-1 expression is associated with tumor antigen-specific CD8+ T cell dysfunction in melanoma patients. J Exp Med. (2010) 207:2175–86. doi: 10.1084/jem.20100637 20819923 PMC2947081

[60] Woo S Turnis M Goldberg M Bankoti J Selby M Nirschl C . Immune inhibitory molecules LAG-3 and PD-1 synergistically regulate T-cell function to promote tumoral immune escape. Cancer Res. (2012) 72:917–27. doi: 10.1158/0008-5472.can-11-1620 22186141 PMC3288154

[61] Shekari N Shanehbandi D Baghbani E Safaei S Masoumi J Baradaran B . VSIG-3/IGSF11 silencing in A2058 melanoma cells simultaneously suppresses melanoma progression and induces anti-tumoral cytokine profile in human T cells: In silico and *in vitro* study. Naunyn Schmiedebergs Arch Pharmacol. (2025) 398:3861–80. doi: 10.1007/s00210-024-03491-z 39365310

[62] Chanmee T Ontong P Konno K Itano N . Tumor-associated macrophages as major players in the tumor microenvironment. Cancers Basel. (2014) 6:1670–90. doi: 10.3390/cancers6031670 25125485 PMC4190561

[63] Hartley G Chow L Ammons D Wheat W Dow S . Programmed cell death ligand 1 (PD-L1) signaling regulates macrophage proliferation and activation. Cancer Immunol Res. (2018) 6:1260–73. doi: 10.1158/2326-6066.cir-17-0537 30012633

[64] Liu Y Zugazagoitia J Ahmed F Henick B Gettinger S Herbst R . Immune cell PD-L1 colocalizes with macrophages and is associated with outcome in PD-1 pathway blockade therapy. Clin Cancer Res. (2020) 26:970–7. doi: 10.1158/1078-0432.ccr-19-1040 31615933 PMC7024671

[65] Singhal S Stadanlick J Annunziata M Rao A Bhojnagarwala P O’Brien S . Human tumor-associated monocytes/macrophages and their regulation of T cell responses in early-stage lung cancer. Sci Transl Med. (2019) 11. doi: 10.1126/scitranslmed.aat1500 30760579 PMC6800123

[66] Spranger S Bao R Gajewski T . Melanoma-intrinsic β-catenin signalling prevents anti-tumour immunity. Nature. (2015) 523:231–5. doi: 10.1038/nature14404 25970248

[67] Joyce J Fearon D . T cell exclusion, immune privilege, and the tumor microenvironment. Science. (2015) 348:74–7. doi: 10.1126/science.aaa6204 25838376

[68] Lou Y Diao L Cuentas E Denning W Chen L Fan Y . Epithelial-mesenchymal transition is associated with a distinct tumor microenvironment including elevation of inflammatory signals and multiple immune checkpoints in lung adenocarcinoma. Clin Cancer Res. (2016) 22:3630–42. doi: 10.1158/1078-0432.ccr-15-1434 26851185 PMC4947453

[69] Hugo W Zaretsky J Sun L Song C Moreno B Hu-Lieskovan S . Genomic and transcriptomic features of response to anti-PD-1 therapy in metastatic melanoma. Cell. (2016) 165:35–44. doi: 10.1016/j.cell.2016.02.065 26997480 PMC4808437

[70] Vesely M Zhang T Chen L . Resistance mechanisms to anti-PD cancer immunotherapy. Annu Rev Immunol. (2022) 40:45–74. doi: 10.1146/annurev-immunol-070621-030155 35471840

[71] Mandalà M Tondini C Merelli B Massi D . Rationale for new checkpoint inhibitor combinations in melanoma therapy. Am J Clin Dermatol. (2017) 18:597–611. doi: 10.1007/s40257-017-0282-0 28432648

[72] Huang R Francois A McGray A Miliotto A Odunsi K . Compensatory upregulation of PD-1, LAG-3, and CTLA-4 limits the efficacy of single-agent checkpoint blockade in metastatic ovarian cancer. Oncoimmunology. (2017) 6:e1249561. doi: 10.1080/2162402x.2016.1249561 28197366 PMC5283642

[73] Koyama S Akbay E Li Y Herter-Sprie G Buczkowski K Richards W . Adaptive resistance to therapeutic PD-1 blockade is associated with upregulation of alternative immune checkpoints. Nat Commun. (2016) 7:10501. doi: 10.1038/ncomms10501 26883990 PMC4757784

[74] Liu J Yuan Y Chen W Putra J Suriawinata AA Schenk AD . Immune-checkpoint proteins VISTA and PD-1 nonredundantly regulate murine T-cell responses. Proc Natl Acad Sci. (2015) 112:6682–7. doi: 10.1073/pnas.1420370112 25964334 PMC4450438

[75] Wright Q Gonzalez CJ Wells J Leggatt G . PD-1 and beyond to activate T cells in cutaneous squamous cell cancers: the case for 4-1BB and VISTA antibodies in combination therapy. Cancers Basel. (2021) 13. doi: 10.3390/cancers13133310 34282763 PMC8269268

[76] Zhou Q Munger M Veenstra R Weigel B Hirashima M Munn D . Coexpression of Tim-3 and PD-1 identifies a CD8+ T-cell exhaustion phenotype in mice with disseminated acute myelogenous leukemia. Blood. (2011) 117:4501–10. doi: 10.1182/blood-2010-10-310425 21385853 PMC3099570

[77] Liu Y Cai P Wang N Zhang Q Chen F Shi L . Combined blockade of Tim-3 and MEK inhibitor enhances the efficacy against melanoma. Biochem Biophys Res Commun. (2017) 484:378–84. doi: 10.1016/j.bbrc.2017.01.128 28132803

[78] Maruhashi T Sugiura D Okazaki I Shimizu K Maeda T Ikubo J . Binding of LAG-3 to stable peptide-MHC class II limits T cell function and suppresses autoimmunity and anti-cancer immunity. Immunity. (2022) 55:912–924.e8. doi: 10.1016/j.immuni.2022.03.013 35413245

[79] Jiang H Ni H Zhang P Guo X Wu M Shen H . PD-L1/LAG-3 bispecific antibody enhances tumor-specific immunity. Oncoimmunology. (2021) 10:1943180. doi: 10.1080/2162402x.2021.1943180 34239776 PMC8237984

[80] Ohta A Kini R Ohta A Subramanian M Madasu M Sitkovsky M . The development and immunosuppressive functions of CD4(+) CD25(+) FoxP3(+) regulatory T cells are under influence of the adenosine-A2A adenosine receptor pathway. Front Immunol. (2012) 3:190. doi: 10.3389/fimmu.2012.00190 22783261 PMC3389649

[81] Willingham S Ho P Hotson A Hill C Piccione E Hsieh J . A2AR antagonism with CPI-444 induces antitumor responses and augments efficacy to anti-PD-(L)1 and anti-CTLA-4 in preclinical models. Cancer Immunol Res. (2018) 6:1136–49. doi: 10.1158/2326-6066.cir-18-0056 30131376

[82] Mariathasan S Turley S Nickles D Castiglioni A Yuen K Wang Y . TGFβ attenuates tumour response to PD-L1 blockade by contributing to exclusion of T cells. Nature. (2018) 554:544–8. doi: 10.1038/nature25501 29443960 PMC6028240

[83] Pitt J Vétizou M Daillère R Roberti M Yamazaki T Routy B . Resistance mechanisms to immune-checkpoint blockade in cancer: tumor-intrinsic and -extrinsic factors. Immunity. (2016) 44:1255–69. doi: 10.1016/j.immuni.2016.06.001 27332730

[84] Zaretsky J Garcia-Diaz A Shin D Escuin-Ordinas H Hugo W Hu-Lieskovan S . Mutations associated with acquired resistance to PD-1 blockade in melanoma. N Engl J Med. (2016) 375:819–24. doi: 10.1056/nejmoa1604958 27433843 PMC5007206

[85] Gilmour C DeLaney E Parthasarathy PB Roy D Ta HM Zakeri A . T cell–intrinsic VISTA expression promotes resistance to CTLA-4 blockade by restricting CD8+ T cell responses. J Clin Invest. (2026) 136:e195668. doi: 10.1172/jci195668 41837284 PMC12987620

[86] Robert C Thomas L Bondarenko I O’Day S Weber J Garbe C . Ipilimumab plus dacarbazine for previously untreated metastatic melanoma. N Engl J Med. (2011) 364:2517–26. doi: 10.1056/nejmoa1104621 21639810

[87] Tawbi H Forsyth P Algazi A Hamid O Hodi F Moschos S . Combined nivolumab and ipilimumab in melanoma metastatic to the brain. N Engl J Med. (2018) 379:722–30. doi: 10.1056/nejmoa1805453 30134131 PMC8011001

[88] Kjeldsen J Lorentzen C Martinenaite E Ellebaek E Donia M Holmstroem R . A phase 1/2 trial of an immune-modulatory vaccine against IDO/PD-L1 in combination with nivolumab in metastatic melanoma. Nat Med. (2021) 27:2212–23. doi: 10.1038/s41591-021-01544-x 34887574 PMC8904254

[89] Ye Y Wang J Hu Q Hochu G Xin H Wang C . Synergistic transcutaneous immunotherapy enhances antitumor immune responses through delivery of checkpoint inhibitors. ACS Nano. (2016) 10:8956–63. doi: 10.1021/acsnano.6b04989 27599066

[90] Sorensen M Holst P Steffensen M Christensen J Thomsen A . Adenoviral vaccination combined with CD40 stimulation and CTLA-4 blockage can lead to complete tumor regression in a murine melanoma model. Vaccine. (2010) 28:6757–64. doi: 10.1016/j.vaccine.2010.07.066 20682365

[91] Tolcher A Sznol M Hu-Lieskovan S Papadopoulos K Patnaik A Rasco D . Phase ib study of utomilumab (PF-05082566), a 4-1BB/CD137 agonist, in combination with pembrolizumab (MK-3475) in patients with advanced solid tumors. Clin Cancer Res. (2017) 23:5349–57. doi: 10.1158/1078-0432.ccr-17-1243 28634283

[92] Roberts D Franklin N Kingeter L Yagita H Tutt A Glennie M . Control of established melanoma by CD27 stimulation is associated with enhanced effector function and persistence, and reduced PD-1 expression of tumor infiltrating CD8(+) T cells. J Immunother. (2010) 33:769–79. doi: 10.1097/cji.0b013e3181ee238f 20842060 PMC2955154

[93] Horvath L Thienpont B Zhao L Wolf D Pircher A . Overcoming immunotherapy resistance in non-small cell lung cancer (NSCLC) - novel approaches and future outlook. Mol Cancer. (2020) 19:141. doi: 10.1186/s12943-020-01260-z 32917214 PMC7488475

[94] Skeate J Otsmaa M Prins R Fernandez D Da SD Kast W . TNFSF14: LIGHTing the way for effective cancer immunotherapy. Front Immunol. (2020) 11:922. doi: 10.3389/fimmu.2020.00922 32499782 PMC7243824

[95] Jia L Pang M Fan M Tan X Wang Y Huang M . A pH-responsive Pickering Nanoemulsion for specified spatial delivery of Immune Checkpoint Inhibitor and Chemotherapy agent to Tumors. Theranostics. (2020) 10:9956–69. doi: 10.7150/thno.46089 32929327 PMC7481420

[96] Kakavand H Jackett L Menzies A Gide T Carlino M Saw R . Negative immune checkpoint regulation by VISTA: a mechanism of acquired resistance to anti-PD-1 therapy in metastatic melanoma patients. Mod Pathol. (2017) 30:1666–76. doi: 10.1038/modpathol.2017.89 28776578

[97] Flies D Wang S Xu H Chen L . Cutting edge: A monoclonal antibody specific for the programmed death-1 homolog prevents graft-versus-host disease in mouse models. J Immunol. (2011) 187:1537–41. doi: 10.4049/jimmunol.1100660 21768399 PMC3150865

[98] Mehta N Maddineni S Kelly R Lee R Hunter S Silberstein J . An engineered antibody binds a distinct epitope and is a potent inhibitor of murine and human VISTA. Sci Rep. (2020) 10:15171. doi: 10.1038/s41598-020-71519-4 32938950 PMC7494997

[99] Iadonato S Ovechkina Y Lustig K Cross J Eyde N Frazier E . A highly potent anti-VISTA antibody KVA12123 - a new immune checkpoint inhibitor and a promising therapy against poorly immunogenic tumors. Front Immunol. (2023) 14:1311658. doi: 10.3389/fimmu.2023.1311658 38152397 PMC10751915

[100] Hu X Qie C Jiang J Xie X Chen W Liu W . M351-0056 is a novel low MW compound modulating the actions of the immune-checkpoint protein VISTA. Br J Pharmacol. (2021) 178:1445–58. doi: 10.1111/bph.15357 33450048 PMC9328666

[101] Xiao Y Li Z Zhong N Cao L Liu B Bu L . Charting new frontiers: Co-inhibitory immune checkpoint proteins in therapeutics, biomarkers, and drug delivery systems in cancer care. Transl Oncol. (2023) 38:101794. doi: 10.1016/j.tranon.2023.101794 37820473 PMC10582482

[102] Mitchell M Billingsley M Haley R Wechsler M Peppas N Langer R . Engineering precision nanoparticles for drug delivery. Nat Rev Drug Discov. (2021) 20:101–24. doi: 10.1038/s41573-020-0090-8 33277608 PMC7717100

[103] Moon T Ta H Bhalotia A Paulsen K Hutchinson D Arkema G . Nanoparticles targeting immune checkpoint protein VISTA induce potent antitumor immunity. J Immunother Cancer. (2024) 12. doi: 10.1136/jitc-2024-008977 39209454 PMC11367342

[104] Wright Q Sinha D Wells J Frazer I Gonzalez CJ Leggatt G . Peritumoral administration of immunomodulatory antibodies as a triple combination suppresses skin tumor growth without systemic toxicity. J Immunother Cancer. (2024) 12. doi: 10.1136/jitc-2023-007960 38296598 PMC10831460

[105] van HL Sandin L Moskalev I Ellmark P Dimberg A Black P . Local checkpoint inhibition of CTLA-4 as a monotherapy or in combination with anti-PD1 prevents the growth of murine bladder cancer. Eur J Immunol. (2017) 47:385–93. doi: 10.1002/eji.201646583 27873300

[106] Jiang X Wang J Zheng X Liu Z Zhang X Li Y . Intratumoral administration of STING-activating nanovaccine enhances T cell immunotherapy. J Immunother Cancer. (2022) 10. doi: 10.1136/jitc-2021-003960 35623658 PMC9150169

[107] Rahimian S Fransen M Kleinovink J Amidi M Ossendorp F Hennink W . Polymeric microparticles for sustained and local delivery of antiCD40 and antiCTLA-4 in immunotherapy of cancer. Biomaterials. (2015) 61:33–40. doi: 10.1016/j.biomaterials.2015.04.043 25993015

[108] Sung E Ko M Won J Jo Y Park E Kim H . LAG-3xPD-L1 bispecific antibody potentiates antitumor responses of T cells through dendritic cell activation. Mol Ther. (2022) 30:2800–16. doi: 10.1016/j.ymthe.2022.05.003 35526096 PMC9372323

[109] Hay C Sult E Huang Q Mulgrew K Fuhrmann S McGlinchey K . Targeting CD73 in the tumor microenvironment with MEDI9447. Oncoimmunology. (2016) 5:e1208875. doi: 10.1080/2162402x.2016.1208875 27622077 PMC5007986

[110] Turiello R Capone M Morretta E Monti M Madonna G Azzaro R . Exosomal CD73 from serum of patients with melanoma suppresses lymphocyte functions and is associated with therapy resistance to anti-PD-1 agents. J Immunother Cancer. (2022) 10. doi: 10.1136/jitc-2021-004043 35273100 PMC8915288

[111] Hu Z Zheng B Xu J Gao S Lu W . An albumin-bound drug conjugate of paclitaxel and indoleamine-2,3-dioxygenase inhibitor for enhanced cancer chemo-immunotherapy. Nanotechnology. (2020) 31:295101. doi: 10.1088/1361-6528/ab824d 32203949

[112] Alimohammadi R Mahmoodi CG Alimohammadi M Ghaffari-Nazari H Rahimi A Mortaz E . Dual blockage of both PD-L1 and CD47 enhances the therapeutic effect of oxaliplatin and FOLFOX in CT-26 mice tumor model. Sci Rep. (2023) 13:2472. doi: 10.1038/s41598-023-29363-9 36774400 PMC9922272

[113] Cha J Yang W Xia W Wei Y Chan L Lim S . Metformin promotes antitumor immunity via endoplasmic-reticulum-associated degradation of PD-L1. Mol Cell. (2018) 71:606–620.e7. doi: 10.1016/j.molcel.2018.07.030 30118680 PMC6786495

[114] Zheng X Liu Z Mi M Wen Q Wu G Zhang L . Disulfiram improves the anti-PD-1 therapy efficacy by regulating PD-L1 expression via epigenetically reactivation of IRF7 in triple negative breast cancer. Front Oncol. (2021) 11:734853. doi: 10.3389/fonc.2021.734853 34858816 PMC8631359

[115] Xiao Z Wang D Wang C Chen Z Huang C Yang Y . PEIGel: A biocompatible and injectable scaffold with innate immune adjuvanticity for synergized local immunotherapy. Mater Today Bio. (2022) 15:100297. doi: 10.1016/j.mtbio.2022.100297 35637855 PMC9142629

[116] Claus C Ferrara C Xu W Sam J Lang S Uhlenbrock F . Tumor-targeted 4-1BB agonists for combination with T cell bispecific antibodies as off-the-shelf therapy. Sci Transl Med. (2019) 11. doi: 10.1126/scitranslmed.aav5989 31189721 PMC7181714

[117] Wang Y Deng J Wang L Zhou T Yang J Tian Z . Expression and clinical significance of PD-L1, B7-H3, B7-H4 and VISTA in craniopharyngioma. J Immunother Cancer. (2020) 8. doi: 10.1136/jitc-2019-000406 32958683 PMC7507895

[118] Grob J Gonzalez R Basset-Seguin N Vornicova O Schachter J Joshi A . Pembrolizumab monotherapy for recurrent or metastatic cutaneous squamous cell carcinoma: A single-arm phase II trial (KEYNOTE-629). J Clin Oncol. (2020) 38:2916–25. doi: 10.1200/jco.19.03054 32673170 PMC7460151

[119] Le Mercier I Chen W Lines JL Day M Li J Sergent P . VISTA regulates the development of protective antitumor immunity. Cancer Res. (2014) 74:1933–44. doi: 10.1158/0008-5472.can-13-1506 24691994 PMC4116689

[120] Tang D Zhao L Yan F Ren C Xu K Zhao K . Expression of VISTA regulated via IFN-γ governs endogenous T-cell function and exhibits correlation with the efficacy of CD19 CAR-T cell treated B-malignant mice. J Immunother Cancer. (2024) 12. doi: 10.1136/jitc-2023-008364 38925679 PMC11202651

[121] Gettinger S Choi J Hastings K Truini A Datar I Sowell R . Impaired HLA class I antigen processing and presentation as a mechanism of acquired resistance to immune checkpoint inhibitors in lung cancer. Cancer Discov. (2017) 7:1420–35. doi: 10.1158/2159-8290.cd-17-0593 29025772 PMC5718941

[122] Lepletier A Madore J O’Donnell J Johnston R Li X McDonald E . Tumor CD155 expression is associated with resistance to anti-PD1 immunotherapy in metastatic melanoma. Clin Cancer Res. (2020) 26:3671–81. doi: 10.1158/1078-0432.ccr-19-3925 32345648

[123] Blando J Sharma A Higa M Zhao H Vence L Yadav S . Comparison of immune infiltrates in melanoma and pancreatic cancer highlights VISTA as a potential target in pancreatic cancer. Proc Natl Acad Sci U A. (2019) 116:1692–7. doi: 10.1073/pnas.1811067116 30635425 PMC6358697

[124] Heinhuis K Carlino M Joerger M Di NM Meniawy T Rottey S . Safety, tolerability, and potential clinical activity of a glucocorticoid-induced TNF receptor-related protein agonist alone or in combination with nivolumab for patients with advanced solid tumors: A phase 1/2a dose-escalation and cohort-expansion clinical trial. JAMA Oncol. (2020) 6:100–7. doi: 10.1001/jamaoncol.2019.3848 31697308 PMC6865300

[125] Afzal M Mercado R Shirai K . Efficacy of metformin in combination with immune checkpoint inhibitors (anti-PD-1/anti-CTLA-4) in metastatic Malignant melanoma. J Immunother Cancer. (2018) 6:64. doi: 10.1186/s40425-018-0375-1 29966520 PMC6027578

[126] Cerezo M Tichet M Abbe P Ohanna M Lehraiki A Rouaud F . Metformin blocks melanoma invasion and metastasis development in AMPK/p53-dependent manner. Mol Cancer Ther. (2013) 12:1605–15. doi: 10.1158/1535-7163.mct-12-1226-t 23741061

[127] Eikawa S Nishida M Mizukami S Yamazaki C Nakayama E Udono H . Immune-mediated antitumor effect by type 2 diabetes drug, metformin. Proc Natl Acad Sci U A. (2015) 112:1809–14. doi: 10.1073/pnas.1417636112 25624476 PMC4330733

[128] Yang M Lin C Wang Y Chen K Zhang H Li W . Identification of a cytokine-dominated immunosuppressive class in squamous cell lung carcinoma with implications for immunotherapy resistance. Genome Med. (2022) 14:72. doi: 10.1186/s13073-022-01079-x 35799269 PMC9264601

[129] Jiang P Gu S Pan D Fu J Sahu A Hu X . Signatures of T cell dysfunction and exclusion predict cancer immunotherapy response. Nat Med. (2018) 24:1550–8. doi: 10.1038/s41591-018-0136-1 30127393 PMC6487502

[130] Rozeman E Hoefsmit E Reijers I Saw R Versluis J Krijgsman O . Survival and biomarker analyses from the OpACIN-neo and OpACIN neoadjuvant immunotherapy trials in stage III melanoma. Nat Med. (2021) 27:256–63. doi: 10.1038/s41591-020-01211-7 33558721

[131] Li H Bullock K Gurjao C Braun D Shukla S Bossé D . Metabolomic adaptations and correlates of survival to immune checkpoint blockade. Nat Commun. (2019) 10:4346. doi: 10.1038/s41467-019-12361-9 31554815 PMC6761178

[132] Winograd R Byrne K Evans R Odorizzi P Meyer A Bajor D . Induction of T-cell immunity overcomes complete resistance to PD-1 and CTLA-4 blockade and improves survival in pancreatic carcinoma. Cancer Immunol Res. (2015) 3:399–411. doi: 10.1158/2326-6066.cir-14-0215 25678581 PMC4390506

[133] Formenti S Demaria S . Combining radiotherapy and cancer immunotherapy: a paradigm shift. J Natl Cancer Inst. (2013) 105:256–65. doi: 10.1093/jnci/djs629 23291374 PMC3576324

[134] Starzer A Berghoff A . New emerging targets in cancer immunotherapy: CD27 (TNFRSF7). ESMO Open. (2020) 4:e000629. doi: 10.1136/esmoopen-2019-000629 32152062 PMC7082637

[135] Topalian S Weiner G Pardoll D . Cancer immunotherapy comes of age. J Clin Oncol. (2011) 29:4828–36. doi: 10.1200/jco.2011.38.0899 22042955 PMC3255990

[136] BaSudan A . The role of immune checkpoint inhibitors in cancer therapy. Clin Pr. (2022) 13:22–40. doi: 10.3390/clinpract13010003 36648843 PMC9844484

[137] Zelin E Zalaudek I Agozzino M Dianzani C Dri A Di MN . Neoadjuvant therapy for non-melanoma skin cancer: updated therapeutic approaches for basal, squamous, and merkel cell carcinoma. Curr Treat Options Oncol. (2021) 22:35. doi: 10.1007/s11864-021-00826-3 33725197 PMC7966643

[138] Schultz L Mackall C . Driving CAR T cell translation forward. Sci Transl Med. (2019) 11. doi: 10.1126/scitranslmed.aaw2127 30814337

